# Genome‐wide evolutionary response of European oaks during the Anthropocene

**DOI:** 10.1002/evl3.269

**Published:** 2022-01-05

**Authors:** Dounia Saleh, Jun Chen, Jean‐Charles Leplé, Thibault Leroy, Laura Truffaut, Benjamin Dencausse, Céline Lalanne, Karine Labadie, Isabelle Lesur, Didier Bert, Frédéric Lagane, François Morneau, Jean‐Marc Aury, Christophe Plomion, Martin Lascoux, Antoine Kremer

**Affiliations:** ^1^ UMR BIOGECO, INRAE Université de Bordeaux Cestas 33612 France; ^2^ College of Life Sciences Zhejiang University Hangzhou 310058 China; ^3^ Department of Botany and Biodiversity Research University of Vienna Vienna 1010 Austria; ^4^ Genoscope, Institut de Biologie François Jacob, Commissariat à l’énergie atomique (CEA) Université de Paris‐Saclay Evry 91057 France; ^5^ Helix Venture Merignac 33700 France; ^6^ Département Recherche Développement Innovation Office National des Forêts Boigny‐Sur‐Bionne 45760 France; ^7^ Current Address: Service de l'Information Statistique Forestière et Environnementale Institut National de l'Information géographique et Forestière Nogent‐sur‐Vernisson 45290 France; ^8^ Génomique Métabolique, Genoscope, Institut François Jacob, CEA, CNRS Univ Evry, Université Paris‐Saclay Evry 91057 France; ^9^ Department of Ecology and Genetics, Evolutionary Biology Centre Uppsala University Uppsala SE‐75236 Sweden

**Keywords:** Anthropocene, evolution, linked selection, Little Ice Age, *Quercus petraea*

## Abstract

The pace of tree microevolution during Anthropocene warming is largely unknown. We used a retrospective approach to monitor genomic changes in oak trees since the Little Ice Age (LIA). Allelic frequency changes were assessed from whole‐genome pooled sequences for four age‐structured cohorts of sessile oak (*Quercus petraea*) dating back to 1680, in each of three different oak forests in France. The genetic covariances of allelic frequency changes increased between successive time periods, highlighting genome‐wide effects of linked selection. We found imprints of parallel linked selection in the three forests during the late LIA, and a shift of selection during more recent time periods of the Anthropocene. The changes in allelic covariances within and between forests mirrored the documented changes in the occurrence of extreme events (droughts and frosts) over the last 300 years. The genomic regions with the highest covariances were enriched in genes involved in plant responses to pathogens and abiotic stresses (temperature and drought). These responses are consistent with the reported sequence of frost (or drought) and disease damage ultimately leading to the oak dieback after extreme events. They provide support for adaptive evolution of long‐lived species during recent climatic changes. Although we acknowledge that other sources (e.g., gene flow, generation overlap) may have contributed to temporal covariances of allelic frequency changes, the consistent and correlated response across the three forests lends support to the existence of a systematic driving force such as natural selection.

**Impact Summary**

A highly debated issue today in ecology is how fast evolutionary changes can occur, especially for long‐lived organisms, and if this pace is sufficient to cope with ongoing climatic changes. Climate warming started after the Little Ice Age (LIA), at the mid‐19th century; thus, multicentennial trees offer a unique opportunity to explore microevolution during documented climatic transitions. We implemented a retrospective approach in European white oak populations using whole genome sequences to track traces of selection. We found genome‐wide evidence of parallel linked selection during the late LIA, and a shift of selection during more recent time periods. Our results provide the first evidence that selection has operated on long‐lived trees over short time periods, and will likely continue to do so. Beyond oaks, other woody species sharing similar life history traits and attributes (large standing genetic, polygenicity of fitness, high fecundity, and severe selection screening at the juvenile stage) may as well be prone to linked selection and allow retrospective tracking of evolutionary and adaptive pathways. Such retrospective approaches may improve our understanding of future responses to ongoing climatic changes.

Our ability to forecast the response of species to climate change is limited by our lack of knowledge about the pace of adaptive evolution, particularly in long‐lived species, such as trees. Multicentennial trees in the Northern Hemisphere that are old enough to have experienced the transition from the cold Little Ice Age (LIA; 1450–1850) to the warm Anthropocene (1850–today) (Luterbacher et al. 2004; Corona et al. 2010; Luterbacher et al. 2016; Anchukaitis et al. 2017) offer a unique opportunity to assess the extent of evolutionary responses to this extreme and well‐described environmental challenge. The LIA (Tkachuck 1983) was a cold period characterized by climatic extremes such as long and harsh winters, but also severe droughts that led to plagues, famines, and ultimately revolutions (Pfister 1984; Fagan 2002; Parker 2013). The consequences of the LIA for plants are best illustrated by the recurrent poor crop harvests (Le Roy Ladurie 2004, 2006). Evidence of the impact of the LIA on forest trees is provided by comparisons of tree ring sizes and historical temperature records (Carrer and Urbinati 2006; Edouard et al. 2009). The decrease in temperature during the LIA resulted in a retreat of the forest tree line at high latitudes (Kullman 2005; MacDonald et al. 2008; Linderholm et al. 2014; Kullman 2015; Helama et al. 2020) or altitudes (Camarero et al. 2015) and changes in species composition (Campbell and McAndrews 1993).

Here, we investigated whether climatic trends during and after the LIA and occurrences of extreme events had evolutionary consequences for forest tree populations. We addressed two major questions in this work. First, we investigated whether extreme events occurring during the late LIA left a genomic signature. Second, we investigated whether the shift in climate after the LIA also left a genomic imprint. We chose sessile oak (*Quercus petraea* (Matt.) Liebl.) as the model species for this study, as the oldest forests in Europe contain sessile oak stands that came into existence in the middle of the LIA (near 1650), a time at which the French statesman Colbert implemented even‐aged management in French forests (Gallon 1752). Four age‐structured cohorts of roughly 340, 170, 60, and 12 years old were sampled within three forests, to explore changes in allele frequencies over time. *Quercus petraea* is known to display considerable genetic diversity (Mariette et al. 2002; Kremer and Hipp 2020; Leroy et al. 2020) and to have a high genetic variance for fitness (Alexandre et al. 2020). The selection for viability or the demographic dynamics generated by extreme weather events would be expected to result in changes in allelic frequency.

Climate change‐driven evolution over the course of a few generations has mostly been reported in invasive species (Chown et al. 2015) or in controlled experiments (Ravenscroft et al. 2015). By contrast, in this study, we explored the ability of a native species with high levels of standing genetic variation to respond to recent documented climatic changes. Our approach mirrors experiments monitoring the change in allelic frequencies over successive discrete generations in natura (Malaspinas 2016) or in controlled selection experiments (Schlotterer et al. 2015), except that our study made use of contemporary age‐structured cohorts. Earlier genome‐wide investigations performed with a synchronous approach in common gardens highlighted the multifaceted and unrepeatable signatures of natural selection, characterized by heterogeneous polygenicity (trait architecture determined by a large number of genes with small effects) and allelic heterogeneity (Alberto et al. 2011; Plomion et al. 2016; Rellstab et al. 2016).

In two recent publications, Buffalo and Coop (2019, 2020) showed that allele frequency trajectories can be shifted due to linked selection between selected and neutral loci and generate covariances between allelic frequency changes at successive time periods. Theoretically, the magnitude of covariances depends on the genetic variance of fitness, recombination, and linkage disequilibrium between selected and neutral loci and the strength of selection, whereas the sign of the covariances depends on the maintenance or fluctuation of selection pressures over time. We hypothesized that this approach would be suitable for detecting genomic footprints of selection in the past, during the late LIA, and shifts in selection pressures due to warming after the end of the LIA. We also explored a more qualitative approach addressing the underlying functions of the multiple genes contributing to the covariances of allelic frequency changes. We thus had three objectives: (1) to retrace trends for the covariances of allelic frequencies between age‐structured cohorts spanning the last three centuries in oak stands, (2) to determine whether these trends were repeatable over replicated observations in three different forests, and (3) to explore the gene networks involved in these responses to environmental change.

## Methods

### SAMPLING FORESTS AND AGE STRUCTURED COHORTS

**Figure 1:**
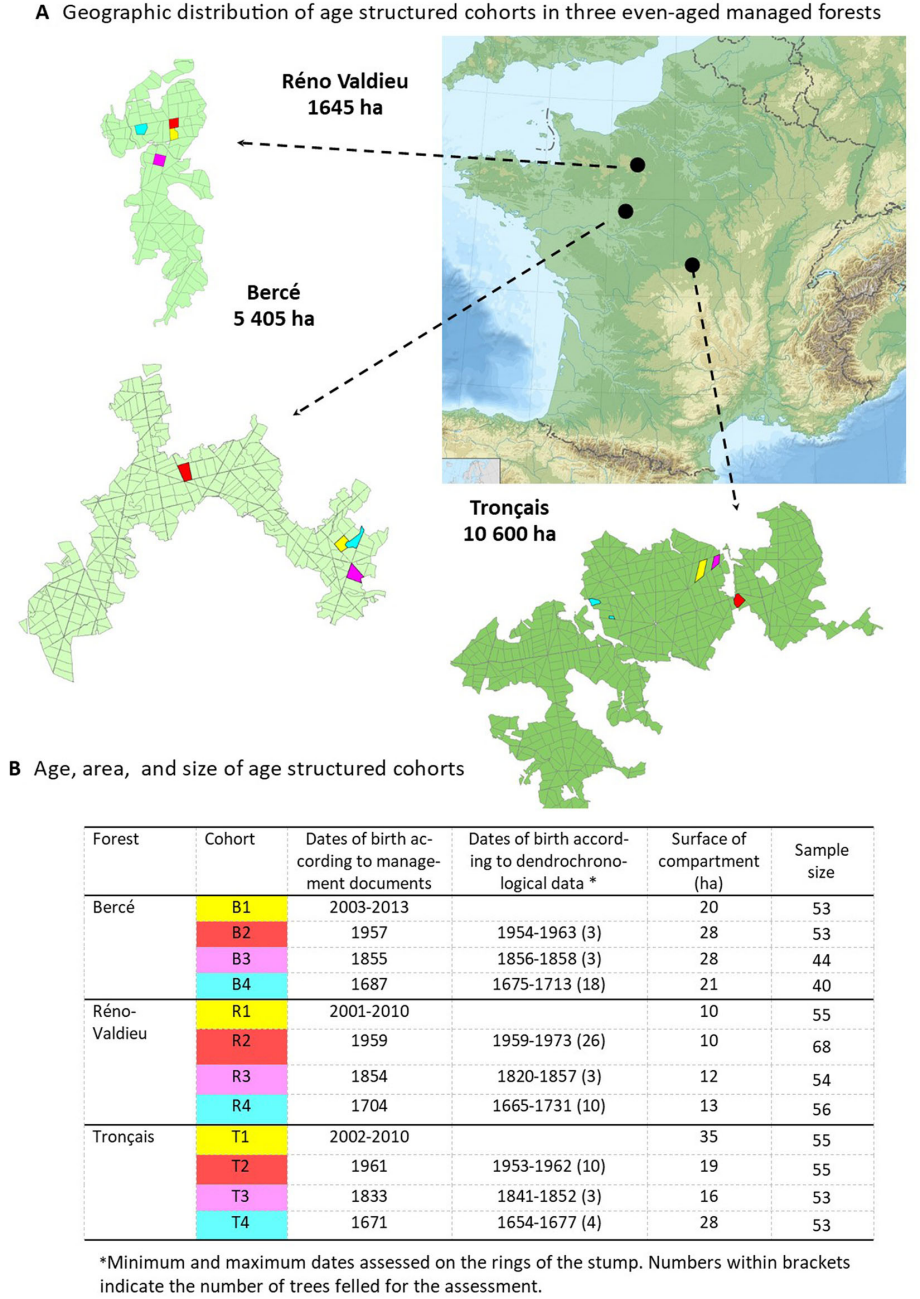
Sampling of forests and age structured cohorts of sessile oak. (A) Distribution of age‐structured cohorts of sessile oak in three even‐aged managed national forests in France. Each forest is subdivided in compartments (about 20 ha in size) limited by the black lines on the forest maps. Age class compartments are evenly distributed in the forests. Densities are extremely high at the seedling stage (>100,0000/ha) and decrease very rapidly due to natural selection during the early stage (≈4000 at age 10). (B) Age, area, and size of age‐structured cohorts. Dendrochronological data of tree rings on felled trees in each cohort allowed to confirm documentary records of tree ages. About 50 trees were randomly sampled in each cohort for whole genome sequencing.

We sampled three oak forests located in the central and western part of France (Bercé, Réno‐Valdieu, and Tronçais; Fig. 1A). These forests include stands of up to 349 years of age when the study started in 2014, and are managed under even‐aged silvicultural regimes (Supporting Information S1). The upper canopy consisted principally of *Quercus petraea*, and historical records and genetic evidence (based on chloroplast DNA haplotypes) indicated that the three forests were of natural origin (Petit et al. 2002). In each forest, we sampled individuals belonging to four age‐class cohorts corresponding to ages of 340, 170, 60, and 12 years (born approximatively in 1680, 1850, 1960, and 2008) (Fig. 1; Table S1). These cohorts are referred to as cohorts 4, 3, 2, and 1, respectively, below. The regeneration period, during which mature trees mate and the stand is renewed by natural seeding, takes today about 10–20 years, but extended over longer periods in the past (up to 30 years). The age of the trees in a given cohort may, therefore, vary by up to 10–30 years. Cohorts were dated on the basis of management records, together with dendrochronological recordings for a few felled trees within each cohort (Fig. 1B). Knowledge of the demographic dynamics of even‐aged forests is required to identify the periods during which selection was at its strongest. When a stand is renewed by natural seeding, a very dense cohort of seedlings develops (more than 100,000 seedlings/ha), the number of plants gradually decreasing to about 4000/ha by the age of 10 years, as a result of natural selection and chance events. Crucially, subsequent silvicultural thinning is applied to only the remaining 4% of the trees. Hence, an oak stand that is about 340 years old today probably underwent its strongest bout of natural selection in the late 17th century, when it was at the seedling stage. Such reasoning provided the rationale for sampling age‐structured cohorts in even‐aged stands for retrospective monitoring of the selective impact of past climatic changes. The different cohorts within a given forest are derived from the same founding population established more than 10,000 years ago (Giesecke 2016; Giesecke and Brewer 2018), but there is no direct traceable generation‐to‐generation link between the cohorts. The three forests as well share a common evolutionary history as they all derived from the iberic glacial refugial during the Last Glacial Maximum (Petit et al. 2002). Therefore, from an evolutionary stand point, the three forests cannot be considered as independent replicates.

High‐resolution regional and temporal temperature reconstructions based on a combination of instrumental data (for the most recent period), documentary records, ice core, and tree ring proxy data revealed clear temperature trends over the last 340 years in the three forests (Luterbacher et al. 2004, 2016) (Supporting Information S2 and Figs. S1–S3).

### DNA EXTRACTION AND SEQUENCING

Leaf samples were collected from cohort 1 in the spring and summer 2014, whereas cambium samples were harvested at the base of the trunk with a punch for the three older cohorts. The sample size for each cohort was between 40 and 68 trees (Fig. 1B). DNA was extracted from the 639 samples with a Qiagen extraction kit. DNA yields were measured with a NanoDrop 1000 spectrophotometer (NanoDrop Technologies, Wilmington, DE, USA) and DNA samples were pooled in equimolar amounts within each cohort from each forest. The 12 pools (3 forests × 4 cohorts) were sequenced on an Illumina HiSeq4000 sequencer generating 150 bp paired‐end reads.

### SNP DISCOVERY

The various steps in the SNP calling pipeline were as described in Altmann et al. (2012) and Pfeifer (2017). The adapters and primers were removed and reads were trimmed to remove nucleotides with a quality value below 20 from the two ends. The sequences between the second unknown nucleotide and the end of the read were removed, and reads of less than 30 nucleotides in length were discarded. Finally, the read pairs from the low‐concentration spike‐in Illumina PhiX Control library were removed. We obtained a mean of 348,863,070 reads per pool (Table 1). The reads were mapped onto the version 2.3 *Q. robur* genome assembly (Plomion et al. 2018) with bwa‐mem, with a seed size of 39 (Li and Durbin 2010). Incorrectly paired reads and reads giving multiple alignments were removed with samtools (Li et al. 2009). Duplications were removed with Picard tools (no publication, Broad Institute). Base Alignment Quality (BAQ) was calculated with samtools (Li et al. 2009). Pileup files were generated for each scaffold over all forests, with samtools. These files were converted into synchronized pileups with a minimum alignment quality of 10, and allele frequencies were calculated for SNPs with a minimum count of two for minor alleles, a minimum coverage of 40×, and a maximum coverage of 10% of total coverage within each pool, with Popoolation2 (Kofler et al. 2011). Further filtering was applied to the three types of pileup files, to select biallelic SNPs with a minimum minor allele frequency of 0.02.

**Table 1:** SNP diversity statistics of the age structured cohorts

Forest	Cohort	Number of reads after postprocessing	Number of SNPs	π ± SD
Bercé	B1	427,365,137	13,277,388	0.01202 ± 5 × 10^−5^
	B2	444,637,716	13,533,867	0.01208 ± 5 × 10^−5^
	B3	450,468,871	13,586,680	0.01334 ± 8 × 10^−5^
	B4	439,841,819	13,334,778	0.01447 ± 1 × 10^−6^
Réno‐Valdieu	R1	421,851,747	13,344,026	0.01211 ± 5 × 10^−5^
	R2	443,414,110	13,678,790	0.01256 ± 5 × 10^−5^
	R3	435,807,782	13,518,651	0.01203 ± 5 × 10^−5^
	R4	440,543,051	13,605,834	0.01206 ± 5 × 10^−5^
Tronçais	T1	543,901,014	15,592,854	0.01351 ± 7 × 10^−5^
	T2	440,923,574	13,457,837	0.01208 ± 5 × 10^−5^
	T3	437,816,360	13,704,258	0.01215 ± 5 × 10^−5^
	T4	432,251,274	13,514,365	0.01208 ± 5 × 10^−5^

### DIVERSITY WITHIN AND BETWEEN COHORTS

Genetic diversity was estimated on nonoverlapping genomic windows of 10 kb spanning the whole oak genome. For each pool, we therefore generated new pileup files, including monomorphic sites. To take into account the variance in pool size and coverage among populations, we used the *subsample‐pileup.pl* script from Popoolation2 (Kofler et al. 2011) to target a minimum coverage of 30 at each position and for each pool. These parameters were chosen after optimization to minimize the amount of missing data between pools. Tajima's *π* (Tajima 1989) was then calculated with the *variance‐sliding.pl* script. As for the SNP sets, sites with a minimum alignment quality of 10 were retained, and were considered polymorphic if at least two copies of the minor allele were detected among all reads. Windows with a minimum covered fraction of 50% were retained for the calculation of Tajima's *π*. *F*_ST_ values were calculated for each SNP, between each pair of cohorts, using Popoolation2 (Kofler et al. 2011), and were averaged over the whole genome.

### TEMPORAL COVARIANCES

Buffalo and Coop (2019, 2020) have developed a method for testing for genomic signals of selection on polygenic traits due to linked selection, based on temporal covariances of allelic frequency changes. Allele frequency changes were calculated for different time spans separating the cohorts in the three replicated forests. We used the CVTK software package available from http://github.com/vsbuffalo/cvtk to calculate the temporal covariances between allelic frequency changes (Buffalo and Coop 2020). Covariances were standardized by sample heterozygosity.

We filtered SNPs by removing sites with a depth below the number of alleles sampled for each cohort, and with a minor allele frequency below 0.02. Contigs were also filtered out when shorter than 200 kb long (excluding “N”s). Read depth and sample size were used to correct for bias in variance estimates (Buffalo and Coop 2020). Corrections were also done for bias caused by sampling noise common to adjacent time points. This bias stems from the allele frequency at a given time point common to the terms of the covariances between adjacent time periods (e.g., for $\text{Cov}(\Delta_{1680-1850},\;\Delta_{1850-1960})$, allele frequency at 1850 is included in both terms).

Genome‐wide temporal covariances were then computed between pairs of nonoverlapping time spans within each forest. Covariances were calculated based on all SNPs, and for all three forests separately. The mean and 95% confidence intervals of the covariance were obtained by bootstrapping, with 5000 iterations.

The change in the variance of allelic frequency changes over the whole period, from 1680 to 2008, becomes:

(1) $$\begin{array}{ccc} V\;\Delta_{1680-2008}\; & = & \;V\Delta_{1680-1850}+V\Delta_{1850-1960}+V\Delta_{1960-2008}\\ & & +\;2\text{Cov}(\Delta_{1680-1850},\Delta {\;}_{1850-1960})\\ & & +\;2\text{Cov}(\Delta_{1680-1850},\Delta_{1960-2008})\\ & & +\;2\text{Cov}(\Delta_{1850-1960},\Delta_{1960-2008}). \end{array}$$

The contribution of the temporal covariances to $V\Delta_{1680-2008}$ due to linked selection thus becomes (G equation [1] of Buffalo and Coop (2020)):

(2) $$\begin{array}{c} G\left(\Delta_{1680-2008}\right)\;=\frac{2\text{Cov}\left(\Delta_{1680-1850},\Delta {\;}_{1850-1960}\right)+2\text{Cov}\left(\Delta_{1680-1850},\Delta_{1960-2008}\right)+2\text{Cov}\left(\Delta_{1850-1960},\Delta_{1960-2008}\right)}{\;V\Delta_{1680-2008}}.\; \end{array}$$

G estimates the contribution of linked selection to the variance of the allelic frequency changes from the starting point to the most recent time period. Buffalo and Coop (2020) suggested that G can also be understood as the decrease in neutral diversity due to linked selection.

We extended this approach to the calculation of covariances between pairs of forests, (Cov Δ*X_i_*_–_ *_j_*, Δ*Y_i_*_–_ *_j_*) over the same period (*i*–*j*) or at different nonoverlapping time periods. The maintenance of a positive covariance would indicate similar responses and directions of selection (parallel selection) in the different forests, whereas negative values would provide evidence of differences in the direction of selection. The extension to different forests made it possible to estimate covariance over contemporary time periods, or disconnected time scales (adjacent or distant), thereby making it possible to determine whether parallel selection occurred over the same time period, or whether fluctuating selection occurred over different time periods.

### GENE ANNOTATION AND ONTOLOGY ENRICHMENT ANALYSIS

We divided the oak genome into tiles of 100,000 bp in length and calculated temporal variances and covariances for all tiles. Genomic regions with strong responses to linked selection were identified on the basis of covariance values between time intervals above 0.01 for all three replicated forests.

The genes located in the regions with the highest covariances were annotated with various bioinformatic tools. *Arabidopsis thaliana* was initially used as a reference. Orthologous genes between oak and *A. thaliana* were identified for each protein sequence (file “Qrob_PM1N_CDS_aa_20161004.fa.gz,” available at https://urgi.versailles.inra.fr/download/oak/) by selecting the best blast hit (blastP, pvalue < 1e+05) to represent the oak gene model, and *Arabidopsis* GO terms from TAIR were used for annotation (https://www.arabidopsis.org/). For oak genes without orthologs in *Arabidopsis*, we performed eggNOG‐mapper version 2 functional annotation based on fast orthology assignments, using precomputed eggNOG version 5.0 clusters and phylogenies (Huerta‐Cepas et al. 2017, 2019). Gene ontology (GO) terms were inferred from eggNOG orthologous groups (OGs).

Gene sets associated with Biological Processes (BP), Molecular Functions (MF), and Cellular Components (CC) were tested for GO terms enrichment with the “topGO” R package and the “weight” algorithm associated with a Fisher's exact test to select the most relevant terms (Alexa et al. 2006; Alexa and Rahnenfuhrer 2020). A *P*‐value < 0.05 was applied for the statistical test, and no FDR was calculated because the *P*‐values returned by the “weight” method are interpreted by Alexa and Rahnenfuhrer (2020) as corrected or not affected by multiple testing. In addition to the annotation and enrichment analysis, we implemented a more integrated approach by exploring the biological relationships and interactions between genes and biological processes. This step made use of Pathway Studio™ Plant that assembles knowledgebase coming mostly from model plant species but using here oak gene *Arabidopsis* homologs of oak genes as gene entries.

## Results

### DIVERSITY AND DIFFERENTIATION WITHIN AND BETWEEN AGE‐STRUCTURED COHORTS

**Figure 2:**
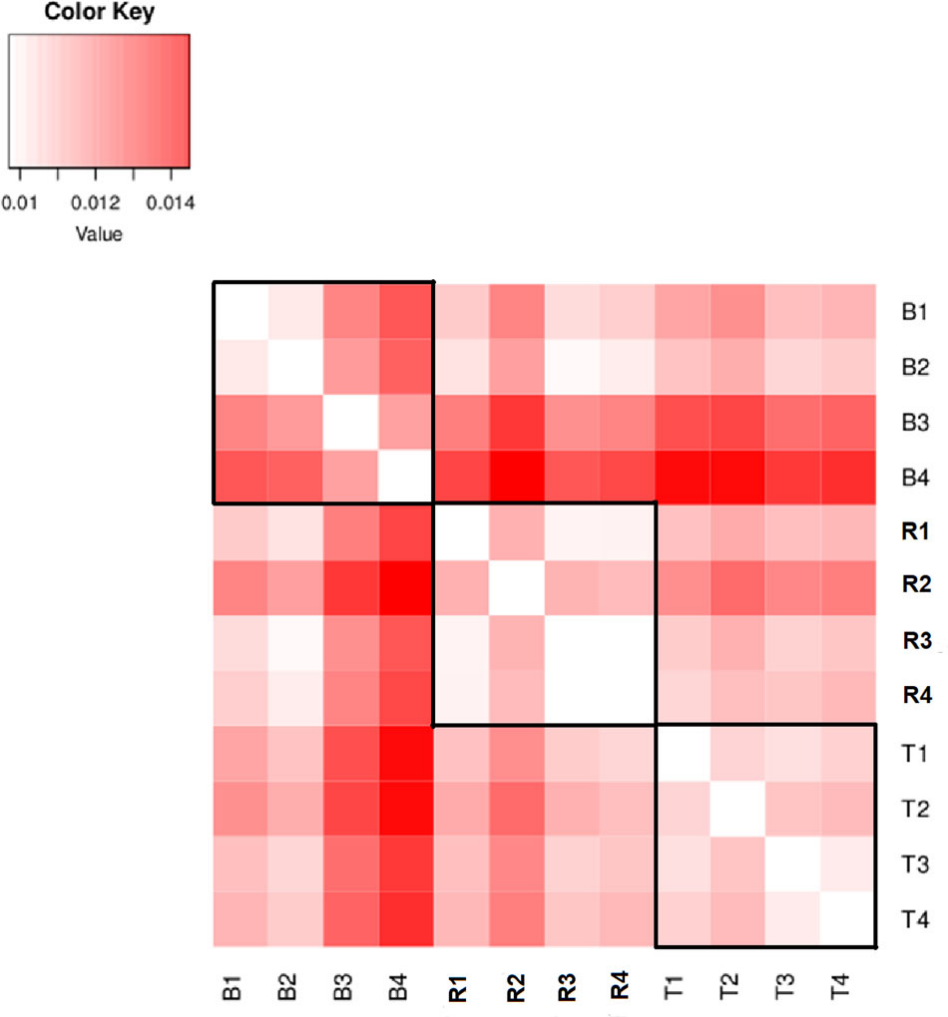
*F*_ST_ values between age‐structured cohorts in the three forests (B: Bercé; R: Réno‐Valdieu; T: Tronçais). Subscripts to forest acronyms indicate the ages of the cohorts: 4, age ∼340 or year of birth ∼1680; 3, age ∼170 or year of birth ∼1850; 2, age ∼60 year of birth ∼1960; 1, age ∼12 or year of birth ∼2008.

On average, more than 13 million SNPs were called in each cohort (Table 1). Within age‐structured cohorts, nucleotide diversity was high (*π* ∼ 0.01205). Levels of diversity were similar across cohorts and forests and consistent with previous estimates for oak (Plomion et al. 2018). The mean pairwise *F*_ST_ between cohorts ranged from 0.010 to 0.015, with no detectable structure between cohorts and forests (Fig. 2).

### TEMPORAL COVARIANCES OF ALLELIC FREQUENCY CHANGES WITHIN FORESTS

**Figure 3:**
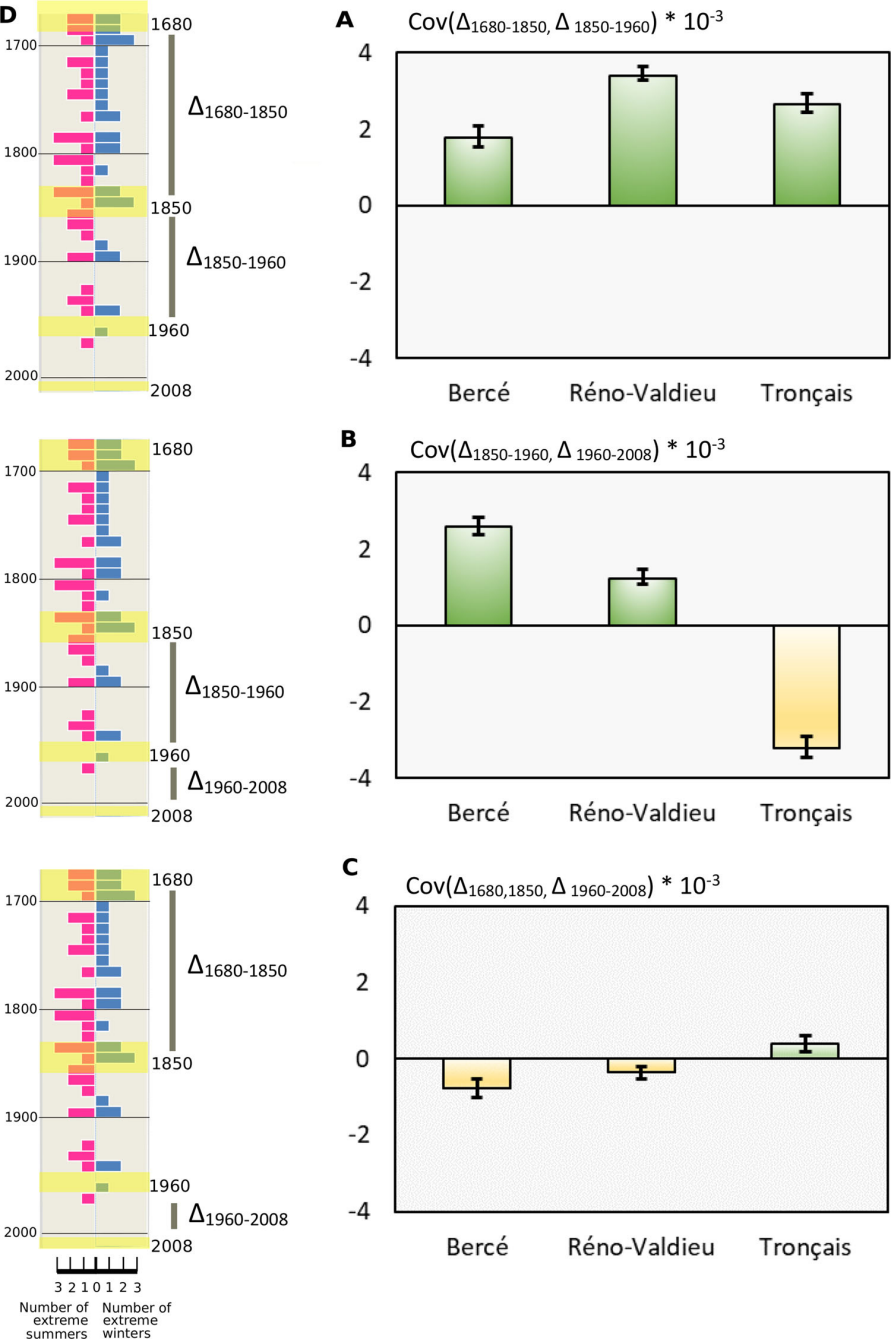
Temporal covariances of allelic frequency changes between different time periods and occurrences of extreme climatic events since the Little Ice Age. Mean and 95% confidence intervals of the covariances were obtained by bootstrapping with 5000 iterations. (A) Temporal covariances of allelic frequency changes between 1680–1850 and 1850–1960 in the three forests. (B) Temporal covariances of allelic frequency changes between 1850–1960 and 1960–2008 in the three forests. (C) Temporal covariances of allelic frequency changes between 1680–1850 and 1960–2008 in the three forests. (D) Timeline subdivided in decades. On the right side of the timeline in blue bars, number of extreme winters per decade according to instrumental temperatures recorded at the Observatory of Paris between 1676 and 2010 (Rousseau 2012) (more details in Fig. S2). On the left side of the timeline in red bars, number of extreme summer droughts per decade according to Cook's data base of Old World megadroughts (Palmer 1965; van der Schrier et al. 2013; Cook et al. 2015) (for more details, see Fig. S3). Highlighted decades in yellow correspond to periods when the cohorts became installed after natural regeneration.

We calculated the covariances of allelic frequency changes between proximal time periods $\left(\text{Cov}(\Delta_{1680-1850},\Delta_{1850-1960}\right)$ and$\;\mathrm{Cov}(\Delta_{1850-1960},\Delta_{1960-2008})$) and distant time periods $\left(\text{Cov}(\Delta_{1680-1850},\Delta_{1960-2008}\right)$). All proximal and distant covariances were significant in all three forests (Fig. 3). The covariances between the earliest adjacent time periods $\text{Cov}(\Delta_{1680-1850},\Delta_{1850-1960})$ were positive and significant in each forest (Fig. 3A). The covariances between the more recent adjacent time periods $\text{Cov}(\Delta_{1850-1960},\Delta_{1960-2008})$ varied between the three forests, being positive in Bercé, slightly lower in Réno‐Valdieu, and negative in Tronçais (Fig. 3B). Finally, the covariances between distant time periods were much lower than those between adjacent time periods in Bercé and Réno‐Valdieu, and also partially in Tronçais (Fig. 3C). Overall, the patterns illustrated in Figure 3 show a shift of covariances between adjacent time periods from the earliest periods considered (1680–1850, 1850–1960) to the more recent time periods (1850–1960, 1960–2008).

**Figure 4:**
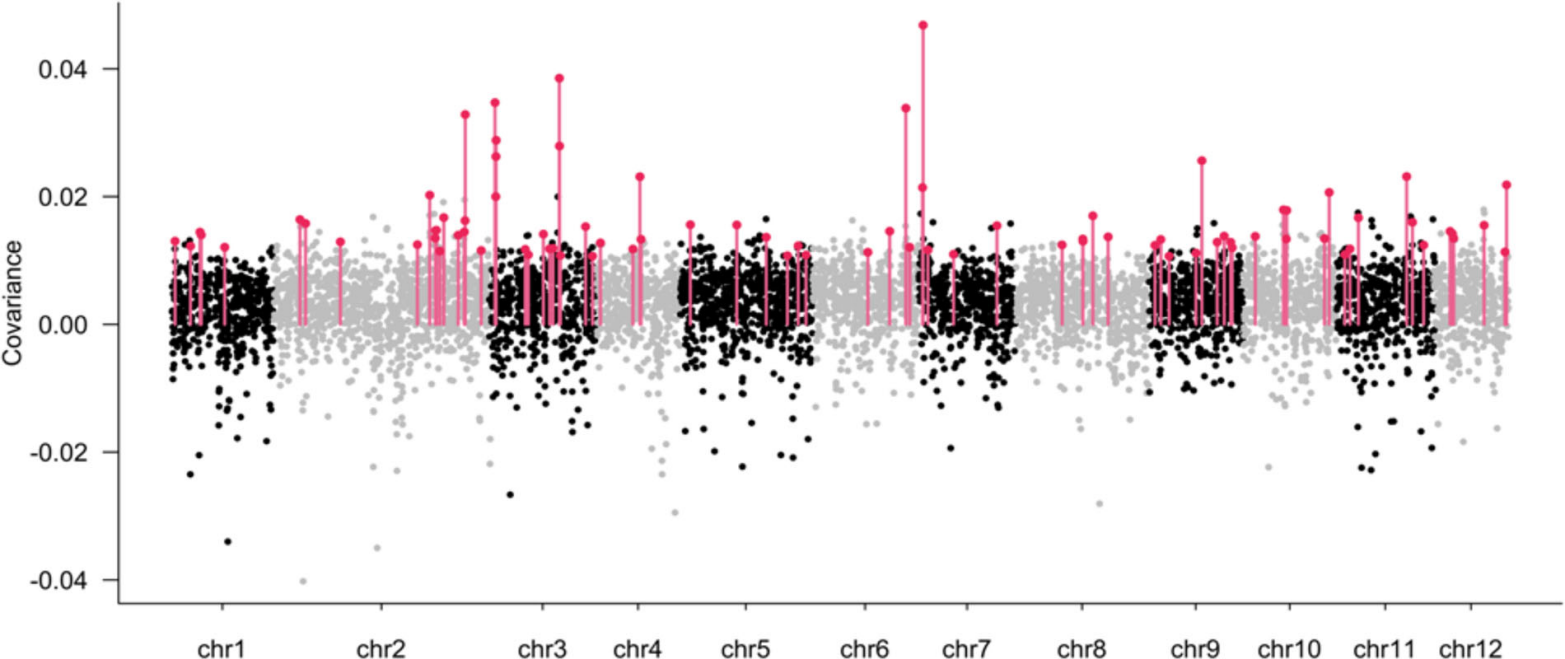
Manhattan plots of the temporal covariances of allelic frequency changes between the two oldest time periods (Cov(Δ_1680‐1850_, Δ_1850‐1960_)) calculated at the tile level, over the whole genome. Black dots correspond to the mean covariance over the three forests. Outliers tiles (red bars) are tiles for which covariances are larger than 0.01 in each of the three forests.

We also estimated the contribution of the temporal covariances to $V\Delta_{1680-2008}$ by calculating $G\Delta_{1680-2008}$ (eq. 2; Table 2). The proportion of the variance of allelic frequency change due to linked selection increased from 1680 to 2008, due to the positive covariances in Bercé (9–16%) and Réno Valdieu (18–22%). It decreased in Tronçais from 12% to −1% (Table 2), due to the overall decrease in covariances between distant time periods. The largest contribution was that of the covariances for the earliest periods $\text{Cov}(\Delta_{1680-1850},\Delta_{1850-1960})$ that were positive in all three forests, resulting in GΔ_1680–1960_ values of 9%, 18%, and 12%, respectively. We explored the genomic distribution of the temporal covariances between the two earliest adjacent periods by calculating the covariances for tiles of 100 Kb and plotting their distribution as Manhattan plots along chromosomes (Fig. 4). Although the overall pattern depicted a rather random distribution, there were a few islands of higher values (especially on chromosomes 2, 3, and 7; Figs. 4 and S5) that led us to investigate the gene composition within these tiles (paragraph GENE ONTOLOGY AND ENRICHMENT ANALYSIS). However, the overall distribution of mean covariances over the three forests across all tiles showed a clear bell‐shaped curve shifted toward positive values, supporting a genome‐wide contribution to the covariances (blue shaded distribution on Fig. S4). These positive covariances exhibit a genome‐wide distribution across all chromosomes (Figs. 4 and S5).

**Table 2:** Contribution of the covariances between allelic frequency changes to the variance of allelic frequency changes between two time points

	Bercé	Réno‐Valdieu	Tronçais
G Δ_1680‐1960_	0.085	0.179	0.119
G Δ_1680‐2008_	0.163	0.221	–0.009

*Note*: G Δ_1680‐1960_: contribution of temporal covariances to the variance of allelic frequency changes from 1680 to 1960. G Δ_1680‐1850_ equals 0 as there is no covariance for the first time period.

### TEMPORAL COVARIANCES OF ALLELIC FREQUENCY CHANGES BETWEEN FORESTS

**Figure 5:**
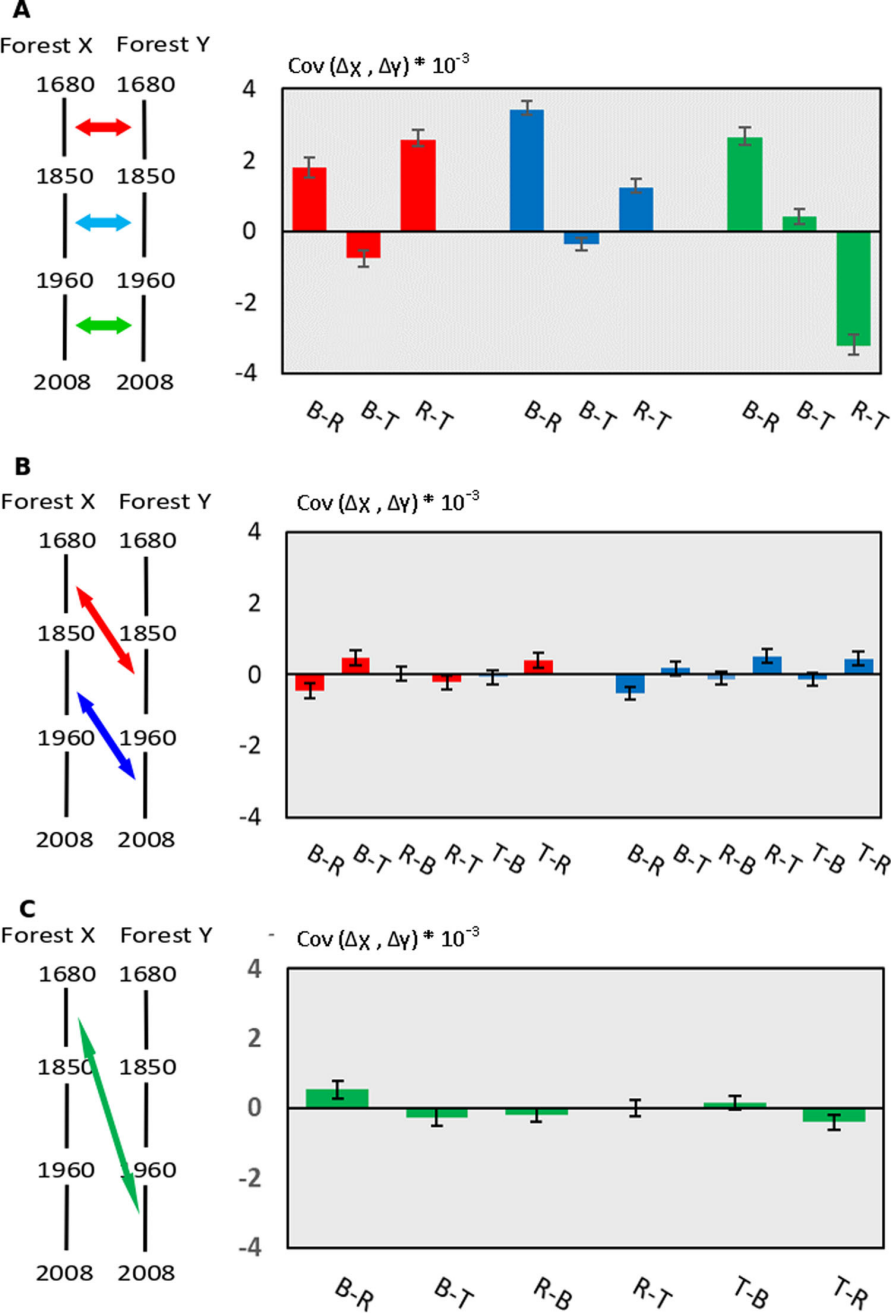
Temporal covariances of allelic frequency changes between the different forests for different time periods. Mean and 95% confidence intervals of the covariances were obtained by bootstrapping with 5000 iterations. Colors of the arrows on the left diagram indicate the time periods considered in the graphs. B: Bercé, R: Réno‐Valdieu, T: Tronçais. (A) Temporal covariances of allelic frequency changes between forests for contemporary time periods. (B) Temporal covariances of allelic frequency changes between forests for adjacent time periods. (C) Temporal covariances of allelic frequency changes between forests for distant time periods.

Significant positive temporal covariances between forests would indicate parallel linked selection. Considering different time periods for calculating the covariances can provide an indication as to when parallel selection occurred. Does it occur at contemporary or distant time periods? We therefore calculated the covariances of allelic frequency changes between the three forests for three different time periods (Fig. 5):

- Contemporary time periods (Fig. 5A);
- Adjacent time periods (Fig. 5B);
- Distant time periods (Fig. 5C).

We did not consider overlapping time periods to avoid the inflation of covariances due to allelic frequency changes common to the covariance terms.

Overall, covariances between forests remained positive and significant for contemporary time periods, particularly between Bercé and Réno‐Valdieu and between Réno‐Valdieu and Tronçais (Fig. 5A). Covariances between Bercé and Tronçais were close to zero regardless of the time period considered. Leaving these latter covariances aside, there was a trend over time toward a decrease in contemporary covariances from more ancient to more recent time periods (Fig. 5A). The covariances between two forests, between adjacent or distant time periods, were strikingly different from contemporary covariances, as they were all close to zero (Fig. 5B, C).

### GENE ONTOLOGY AND ENRICHMENT ANALYSIS

**Figure 6:**
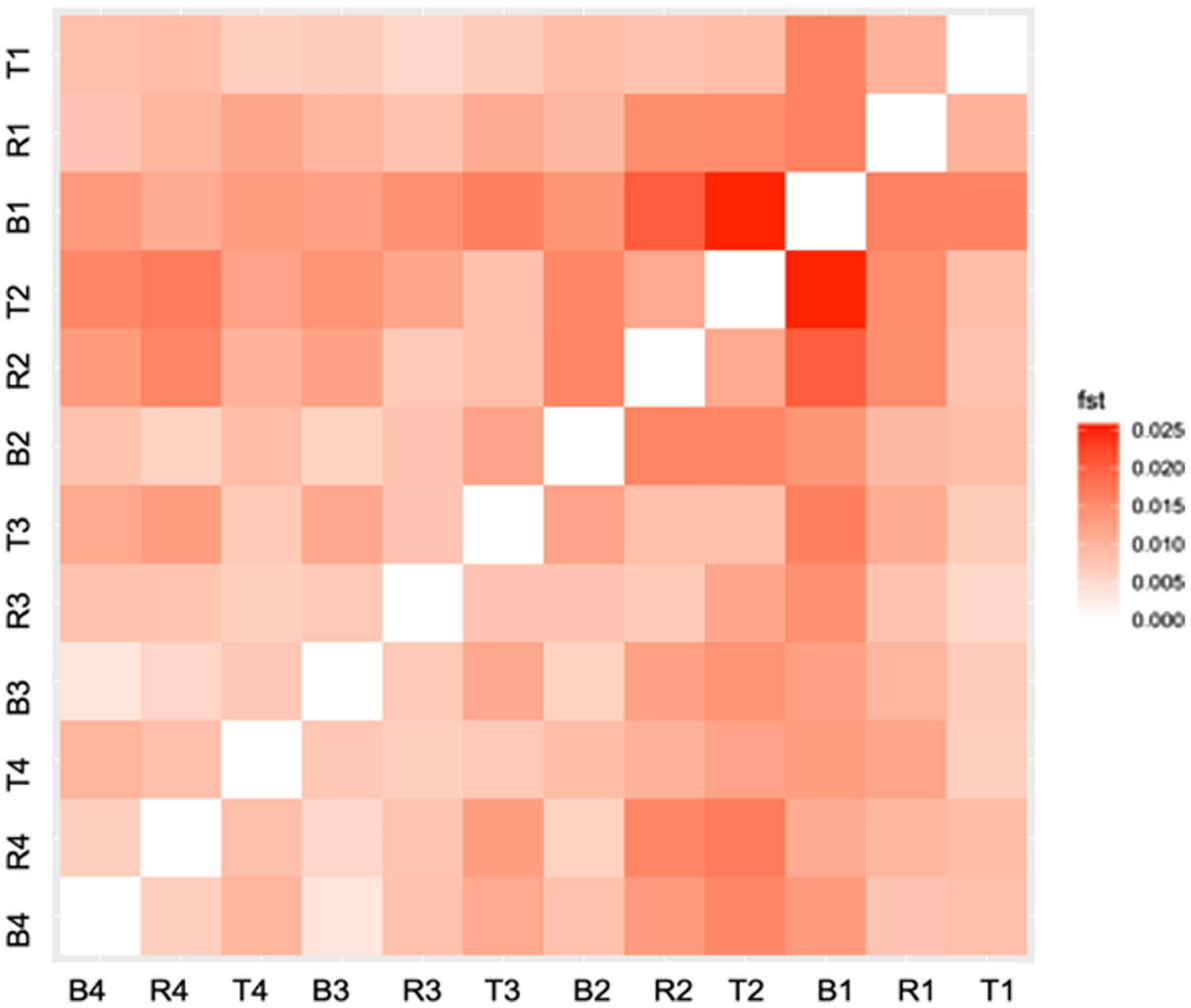
Matrix of *F*_ST_ values of SNPs located in the outlier tiles between all pairs of age‐structured cohorts.

We used the genomic distribution of covariances at the 100 kb tile level (Manhattan plot; Fig. 4) to identify putative regions (tiles) under linked selection. We restricted our analysis to the two earliest time intervals (yielding positive values in all three forests (Fig. 3A), for which strong covariances were also observed between forests (Fig. 5). Tiles exhibiting covariances between the two earliest time intervals $\text{Cov}(\Delta_{1680-1850},\Delta_{1850-1960})$ larger than 0.01 in all three replicates were considered as outliers (open bar distribution on Fig. S4). In total, we identified 104 tiles exhibiting temporal covariances above the threshold, which corresponded to 1% of the tiles (Fig. S4). The outlier tiles were distributed across all chromosomes as shown by the Manhattan plots (Fig. 4). Interestingly, outlier tiles that exhibited the highest covariances between the oldest time periods (Cov(Δ_1680‐1850_, Δ_1850‐1960_)) comprise SNPs that show temporal patterns of differentiation. Indeed, *F*_ST_ values of SNPs between proximal older time periods are larger than between proximal recent time periods, within and between forests, and they constantly decrease from older to recent time periods (Fig. 6). The *F*_ST_ values of those SNPs exhibited temporal patterns that parallel the covariance temporal pattern (Fig. 3). In a few cases (e.g., on chromosomes 2, 3, and 7), we found outlier tiles exhibiting systematically higher values in the three forests (Fig. S5). However, the comparisons of the covariances of the outlier tiles with their *F*_ST_ values (between cohorts delimiting the two earliest time periods) indicate that covariances build up as a result of repeated numerous subtle allelic frequency changes over two successive time periods, rather than by a few recurrent extreme shifts (albeit with a few exceptions in Réno‐Valdieu; Fig. S5).

We inventoried 280 protein coding genes in these regions. Functional annotations of the 280 genes putatively under linked selection are summarized in Table S2. Briefly, out of these 280 genes, 248 received a GO annotation from *A. thaliana*, and two also received eggNOG GO terms, leaving 30 genes without a GO annotation.

**Figure 7:**
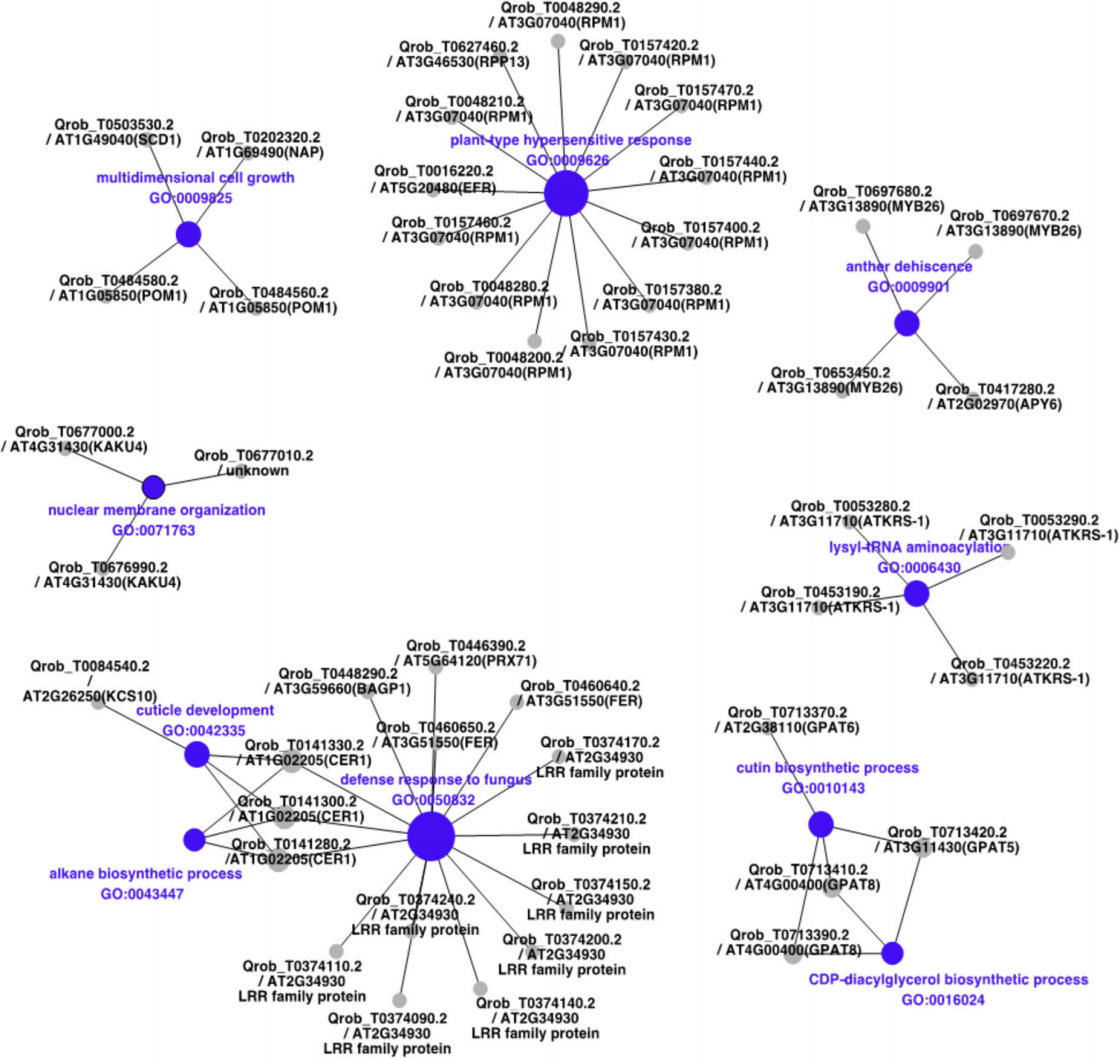
Network plot of the 10 most significant Biological Process (BP terms from gene ontology enrichment analysis). The size of the nodes is proportional to their degree of connectivity. The labels correspond to the name of the *Quercus robur* gene model followed by the locus name of the best *Arabidopsis* homolog and the corresponding *Arabidopsis* gene name (from TAIR: https://www.arabidopsis.org/) between brackets when available. When no gene name exists, a short description (from TAIR or EggNOG databases) is added. If no description is available, the description is set to “unknown.”

The 280 genes revealed significant enrichments in the different gene ontologies (Tables S3–S5). Enrichment analysis identified several Biological Processes (BP), most of which are related to the “plant‐type hypersensitive response,” “defense response to fungus,” “wax and cutin biosynthetic processes,” and “anther dehiscence,” with higher connectivity between the two first terms that gather 15 and 13, genes respectively (Fig. 7; Table S3).

Additional enrichment analysis accounting for other gene ontologies as Molecular Function or Cellular Component and gene network analysis using Pathway Studio™ Plant knowledgebase highlighted as well genes and functions contributing to resistance to biotic or abiotic factors (Supporting Information 3, Fig. S6, and Tables S3–S6).

## Discussion

### TEMPORAL COVARIANCES AS IMPRINTS OF GENOME‐WIDE LINKED SELECTION DURING AND AFTER THE LIA

Temporal allelic frequency changes for genes underlying adaptive polygenic traits have been studied in detail through theoretical approaches (Hollinger et al. 2019; Stephan and John 2020), simulations (Franssen et al. 2017), and empirical case studies (Long et al. 2015). Convergent predictions highlight small and subtle changes for genes targeted by directional selection, and have been supported by multigeneration selection experiments in model organisms (Burke et al. 2010; Barghi et al. 2019; Michalak et al. 2019). Our results suggest that genomic signatures of recent selection are also detected in natural populations of long‐lived nonmodel species and over limited numbers of generations. Rapid evolution was reported at the genomic level in previous studies in nonmodel species in the context of rapid environmental change (Franks et al. 2018; Hamann et al. 2021), strong selection pressures (Dayan et al. 2019), or domestication (Guo et al. 2018), and this process was fueled by the presence of large amounts of standing genetic variation (Bitter et al. 2019; Dayan et al. 2019). The results we obtained in oaks meet theoretical expectations and show that tree populations are under genome‐wide linked selection. Buffalo and Coop (2019) showed that under directional selection (based on an exponential fitness function), the covariances of neutral allele frequency changes over time depend on the genetic variance of fitness (*V*_A_), recombination distance, and linkage disequilibrium. We recently reported that *Q. petraea* forest stands display substantial heritable differences in reproductive success (Alexandre et al. 2020). The genetic variance of fitness in a forest located between two of the forests studied here, Bercé and Réno‐Valdieu, was 0.468 (Alexandre et al. 2020), which is at the upper end of the range of values reported in a recent review of the literature (89% of the reported values were below 0.20) (Hendry et al. 2018). Furthermore, QTL studies of fitness‐related traits in oaks (growth, phenology, and reproduction) have shown that all these traits depend on a large number of loci (Brendel et al. 2008; Derory et al. 2010; Caignard et al. 2019), suggesting the genetic architecture of fitness is highly polygenic. Sessile oak and most likely trees in general are therefore prone to linked selection, provided that environmental constraints are strong enough for selection to operate.

### SHIFTS OF TEMPORAL COVARIANCES MIRROR CLIMATIC TRANSITIONS BETWEEN THE LIA AND MODERN TIMES

If *Q. petraea* populations provide the necessary substrate for linked selection, what were the environmental constraints that triggered viability selection during the time periods considered? There is evidence that the LIA was a period with frequent extreme events in both summer and winter (Dobrovolny et al. 2010; Berchtold et al. 2012; Moreno‐Chamarro et al. 2017) (Figs. S2 and S3). As illustrated in Supporting Information 2, extreme frosts and droughts were more frequent up to the late 19th century than during more recent periods. Frost damage, including tree decline and deaths, was reported after extreme winters in the late LIA. Hausendorff (1940) summarized historical writings about forest districts in Northern Germany mentioning recurrent episodes of adult oak tree mortality during the 1740–1748 period, following the extreme winter of 1739–1740. Many historical documents from Catholic parishes reported extensive tree losses following the winter of 1708–1709, which is known to be the most severe winter in European records (Avila and Avila 1987; Luterbacher et al. 2004). In Eastern France, a historical atlas dating from 1758 reported “The dead material was mainly oak. For example, forty‐six oak high forests belonging to all categories were totally dead.” This high mortality was presumably attributed to the extreme winter of 1708–1709 (Schnitzler 2020). However, the climate improved after 1850. There were only three reported extreme winters (1942, 1947, and 1963) and three extreme droughts (1921, 1934, and 1976) during the 20th century (Figs. S2 and S3), during which oak decline was also recorded (Delatour 1983). These extreme events and the shift in their occurrence over time (during the second half of the 19th century) may be responsible for generating the pattern of linked selection observed in this study.

In even‐aged managed oak stands, selection intensity at the seedlings stage is extremely high and the size of the seedling population very large. Theory (Buffalo and Coop 2019) and empirical data from selection experiments (Buffalo and Coop 2020) suggest that, if the direction of selection is maintained over time, covariances tend to be positive, whereas shifts of directional selection are likely to decrease covariances, possibly resulting in negative values. In experimental selection, in which the direction of selection is maintained over successive generations, temporal covariances are predicted to decay as a result of recombination (Buffalo and Coop 2019). In natural populations, heritable fitness differences, the strength and shifts of direction of selection, and recombination are the drivers of the temporal covariances. The widespread and frequent occurrence of extreme frosts and drought events up to the late 19th century in Europe may therefore be responsible for the positive covariance observed between the two earliest time periods (1680–1850 and 1850–1960; Fig. 3A) within each forest and for the positive covariances at contemporary time scales between forests (Fig. 5A). Conversely, the lower and even negative covariances between the two more recent time periods (1850–1960 and 1960–2008; Fig. 3B) may be a genomic signature of the change in the frequency of extreme events between the mid‐19th century and today and of the shift of directional selection. These observed patterns of temporal covariances were predicted by simulations mimicking different strengths of selection showing that stronger directional selection generates stronger temporal covariances (Buffalo and Coop 2020). The maintenance of positive (or negative) covariances between adjacent time periods is reinforced by the limited impact of recombination in our study case. The short time interval between cohorts would have prevented opportunities for recombination events to break the linked polymorphisms. The time trends for covariances between forests support the interpretation that the strength and shifts of selection since the late LIA have shaped signatures of linked selection. Between‐forest covariances were higher and more consistent across forests during the late LIA, when climatic conditions were harsher than the milder climatic conditions observed in the late 20th century (Fig. 5). Such trends suggest fluctuating selection as also supported by the temporal trends of pairwise *F*_ST_ values of SNPs located in the outlier tiles (Fig. 6).

### BIOTIC INTERACTIONS DRIVEN BY ADAPTIVE RESPONSE TO CLIMATE

The functional analysis highlighted significant enrichments for genes involved mainly in plant defense responses to pathogens, or contributing to abiotic stress responses (temperature and drought) (Fig. 7). Many of the genes encoding resistance proteins (R proteins) belong to the NBS‐LRR families. These R‐genes are widely represented in the oak genome, in which they appear as expanded groups (Plomion et al. 2018). Such NBS‐LRR receptors detect effectors used by pathogens to facilitate infection, and participate in the signaling cascade leading to various responses preventing further infection, such as the hypersensitive response, the production of reactive oxygen species, and cell wall modification (Boyes et al. 1998; Roux 2010; El Kasmi et al. 2017). Interestingly two homologs of genes RPM1 and EFR were both involved in plant defense against *Pseudomonas syringae* (Boyes et al. 1998), which can infect a wide range of herbaceous and woody plants that have suffered frost and freezing damage (Young et al. 1988; Luisetti et al. 1991; Balestra et al. 2009). In addition, five genes encoding beta‐glucosidases that could confer freezing/cold tolerance were identified (Thorlby et al. 2004; Fourrier et al. 2008; Ambroise et al. 2020). These genes may also participate in responses to other biotic/abiotic stresses (Baba et al. 2017; Vassao et al. 2018). Our functional analysis is consistent with the oak responses described in cases of oak decline ultimately leading to the death of the tree. Botanic and pathological descriptions of oak decline during the LIA are lacking, but reports of recurrent sparse oak dieback in more recent decades can be used to retrace the steps leading to oak death following severe winter or drought events. In a review of oak decline in Europe, Thomas et al. (2002) highlighted the combined effects of climatic extremes (drought or frost) and defoliating insects and pathogenic fungi (Thomas et al. 2002). The starting point is an extreme climatic event that weakens the trees (Vanoni et al. 2016), which is then followed by pest and insect attacks, which ultimately kill the tree. For example, episodes of oak decline in the first half of the 20th century in various parts of Europe were caused by a combination of winter frost, summer drought, insect defoliation (caused by oak leaf roller [*Tortrix viridana*] and oak processionary moth *Thaumetopoea processionea*), and several pathogenic fungi (mainly powdery mildew *Erysiphe alphitoides*) and root pathogens (*Armillaria* and *Phytophthora*) (Delatour 1983; Donaubauer 1998; Thomas et al. 2002). In summary, extreme events, such as severe frost or drought, expose trees not only to abiotic stresses, but also to biotic selection pressures. These, in turn, trigger resistance responses, which were identified at genomic level in the functional analysis. We suspect that these processes also operated during the late LIA and that exposure to selection was more stringent, leading more widespread death (Hausendorff 1940; Schnitzler 2020), and, ultimately, to parallel linked selection across the three forests.

### CAVEATS AND LIMITATIONS

The temporal allelic frequency changes assessed in our particular case study of age‐structured cohorts are also potentially subject to other sources of variation that must be considered. Although the theory of temporal covariances generated by linked selection has been developed for discrete generations in isolated populations (Buffalo and Coop 2019), it is unknown how covariances may be shaped in natural settings (Dehasque et al. 2020). We see four (nonexclusive) mechanisms that may have contributed covariances in our study in addition to climate triggered selection: generation overlap, gene flow between cohorts (within the studied forests), gene flow from external populations (not included in our settings), and development‐related changes of fitness. Some of these mechanisms are not independent: gene flow between cohorts reinforces generation overlap and hence their joint effect should be considered. Gene flow may potentially generate positive covariances if the same differentiated populations pollinate repeatedly the studied cohort populations. A realistic scenario to consider in this case is, for example, older cohorts pollinating systematically younger cohorts. This will create a so‐called age‐driven unidirectional gene flow within each forest. The occurrence of age‐driven unidirectional gene flow is minimized in our case because successful matings leading to the recruitment include also pollination from younger trees to the older seed trees in even‐aged managed forests (Supporting Information S1). The large census size of the age classes corresponding to the cohorts, their random spatial distribution (Fig. 1), and the extent of pollen dispersal within forests (Gerber et al. 2014) all reduce the likelihood of age‐driven unidirectional gene flow. Generation overlap may also be enhanced by peculiar demographic scenarios where a limited number of large trees (corresponding in our case to the oldest cohorts) would repeatedly contribute to recruitments to the next generations. In even‐aged managed forests, recruitment is implemented at the level of each compartment, using as seed trees those located in the compartment, thus excluding repeated recruitment from the same seed trees. Repeated unidirectional gene flow from external populations can similarly lead to a covariance signal. For example, consistent pollination from a differentiated population through time could increase covariance. However, highly differentiated populations are unlikely to be present in the study area. Genetic surveys conducted with genome‐wide SNP data show that population differentiation is extremely low (*F*_ST_ values usually lower than 2 %) throughout western lowland Europe (Leroy et al. 2020). Our own data in this study show similar results (Fig. 2): cohorts within forests (and between forests) exhibit extremely low differentiation. The low differentiation is due to limited drift effects and extensive pollen flow as a result of the continuous distribution of *Q. petraea* forests and their large population sizes (also shown by the large π values in Table 1) in this part of the natural distribution. It is thus unlikely that the same “dissimilar” population may have pollinated repeatedly the studied cohorts resulting in positive covariances.

Lastly, the confounding effects of development‐related changes in fitness may have contributed to increases in temporal covariances too. If traits contributing to fitness change over time in a long‐lived species, then genetic covariances of fitness‐related traits will increase between traits assessed at similar ages. Such temporal serial autocorrelations and their decay over time have been reported for growth in pines (Kremer 1992). Such development‐related covariances would be entirely confounded with temporal covariances in our case. However, we would argue that developmental covariances occurring at the adult stage are likely to have a limited impact on temporal covariances, as selection is overwhelmingly more severe at the juvenile stage, with more than 90% of oak seedlings in a stand eliminated before the age of 10 years (Jarret 2004).

Theoretical investigations that have tentatively addressed the combined effects of gene flow, generation overlap, and development‐related variation of fitness are missing. Although we cannot exclude such consequences, we think that they were minimized in our experimental settings. Despite these caveats, and although we acknowledge that the three forests are not evolutionary independent, the similar patterns of temporal covariances observed across the three forests (Figs. 3 and 5) are a strong signal of systematic driving forces operating in the same direction in the three forests. Among the different potential causes that we just discussed, we believe that selection is the most likely one.

## AUTHOR CONTRIBUTIONS

Conception and coordination of the study: AK, CP, and ML. Sampling of forests and cohorts: AK, FM, and LT. Collection of samples and DNA extraction: LT, BD, CL, AK, TL, and DB. Dendrochronological analysis: DB and FL. Whole genome sequencing: JMA, KL, and CP. Bioinformatic analysis: DS, IL, JC, and TL. Enrichment analysis: JCL. Data analysis: DS, JC, AK, and ML. Writing of the manuscript: AK, DS, JC, JCL, ML, and TL. All authors reviewed the manuscript.

## DATA ARCHIVING

The data that support the findings of this study are openly available in the public accessible data repository of INRAE: https://data.inrae.fr/dataset.xhtml?persistentId=doi:10.15454/JLA1A0.

Associate Editor: Z. Gompert

## Supporting information


**Figure S1**. Mean yearly temperature trends in the three studied forests.

**Figure S2**. Number of extreme winters per decade.

**Figure S3**. Number of extreme summer droughts per decade near each studied forest according to Cook's data base of Old World megadroughts (Cook *et al*., 2015).

**Figure S4**. Distribution of the temporal covariances between allelic frequency changes between the two oldest time periods (Cov *(Δ_1680‐1850_, Δ_1850‐1960_))*, calculated for tiles of 100kb.

**Figure S5**. Manhattan plot of the temporal covariances of allelic frequency changes between the two earliest time periods (Cov(Δ_1680‐1850_, Δ_1850‐1960_)) and of *F*st between the cohorts delimiting the time periods (Fst_1680‐1850_ and Fst_1850‐1960_).

**Figure S6** Biological network showing the 15 selected Cell Processes and their 74 connected *Arabidopsis* genes (according to the pathway Studio™Plant database).

**Table S1**. Geographic coordinates of sampled forests and age structured cohorts

**Table S2**: Functional annotation of the 280 oak genes located in outlier tiles

**Table S3**: Significance of Biological Process GO terms according to topGO “weight” method and Fisher's exact test.

**Table S4**: Significance of Molecular Function GO terms according to topGO “weight” method and Fisher's exact test.

**Table S5**: Significance of Cellular Component GO terms according to topGO “weight” method and Fisher's exact test.

**Table S6**: List of the 74 *Arabidopsis* homologs known to participate to at least one cell process with their name used in Pathway Studio™ Plant, their locus name as defined in TAIR (https://www.arabidopsis.org) and alias commonly used in scientific publications

Supporting Information S1, S2, S3
