# Supplementary material for: Genome‐wide evolutionary response of European oaks during the Anthropocene
Source: Evol Lett. 2022 Jan 5;6(1):4–20. doi: 10.1002/evl3.269 (PMC8802238; doi:10.1002/evl3.269)
Supplement: Supplementary file 1 — Figure S1. Mean yearly temperature trends in the three studied forests. [file EVL3-6-4-s009.docx]

**Figure S1**. Mean yearly temperature trends in the three studied forests.

Temperatures were reconstructed according to Luterbacher (Luterbacher *et al*., 2004) using a combination of instrumental data, documentary records and ice core and tree ring proxy data. Yearly means were smoothed over 50 years.

Luterbacher, J., Dietrich, D., Xoplaki, E., Grosjean, M. & Wanner, H. 2004. European seasonal and annual temperature variability, trends, and extremes since 1500. *Science* **303**, 1499-1503
