# Supplementary material for: Genome‐wide evolutionary response of European oaks during the Anthropocene
Source: Evol Lett. 2022 Jan 5;6(1):4–20. doi: 10.1002/evl3.269 (PMC8802238; doi:10.1002/evl3.269)
Supplement: Supplementary file 2 — Figure S2. Number of extreme winters per decade. [file EVL3-6-4-s005.docx]

**Figure S2.** Number of extreme winters per decade.

**Legend:** Data compiled from Instrumental temperatures recorded at the Observatory of Paris between 1676 and 2010 (Rousseau, 2012). Winters were considered as extremes when the mean winter temperature (December-January-February) was lower by more than 2°C to the mean winter temperature during the 19^th^ century in Paris.

Rousseau, D. 2012. in *Canicules et froids extrêmes* (eds J. Berchtold, E. Le Roy Ladurie, J.-P. Sermain, & A. Vasak) 345-360, Hermann, Paris.
