# Supplementary material for: Genome‐wide evolutionary response of European oaks during the Anthropocene
Source: Evol Lett. 2022 Jan 5;6(1):4–20. doi: 10.1002/evl3.269 (PMC8802238; doi:10.1002/evl3.269)
Supplement: Supplementary file 3 — Figure S3. Number of extreme summer droughts per decade near each studied forest according to Cook's data base of Old World megadroughts (Cook et al., 2015). [file EVL3-6-4-s012.docx]

**Figure S3**. Number of extreme summer droughts per decade near each studied forest according to Cook’s data base of Old World megadroughts (Cook *et al*., 2015)..

Summers were considered as extremes when the Palmer’s drought severity index (PDSI), which is a measure of soil moisture availability, was lower than -4 (Palmer, 1965; Van der Schrier et al., 2013). According to Palmer’s classification of drought based on PDSI, values lower than -4 correspond to “extremely dry” conditions, which is also the most extreme class of PDSI.

Cook, E. R. *et al.* 2015. Old World megadroughts and pluvials during the Common Era. *Science Advances* **1**,.

Palmer, W. C. 1965. *Meteorological drought*. Vol. Research paper # 45 (US Department of Commerce, Weather Bureau.

van der Schrier, G., Barichivich, J., Briffa, K. R. & Jones, P. D. 2013 A scPDSI-based global data set of dry and wet spells for 1901-2009. *Journal of Geophysical Research-Atmospheres* **118**, 4025-4048.
