# Supplementary material for: Genome‐wide evolutionary response of European oaks during the Anthropocene
Source: Evol Lett. 2022 Jan 5;6(1):4–20. doi: 10.1002/evl3.269 (PMC8802238; doi:10.1002/evl3.269)
Supplement: Supplementary file 4 — Figure S4. Distribution of the temporal covariances between allelic frequency changes between the two oldest time periods (Cov (Δ1680‐1850, Δ1850‐1960)), calculated for tiles of 100kb. [file EVL3-6-4-s006.docx]

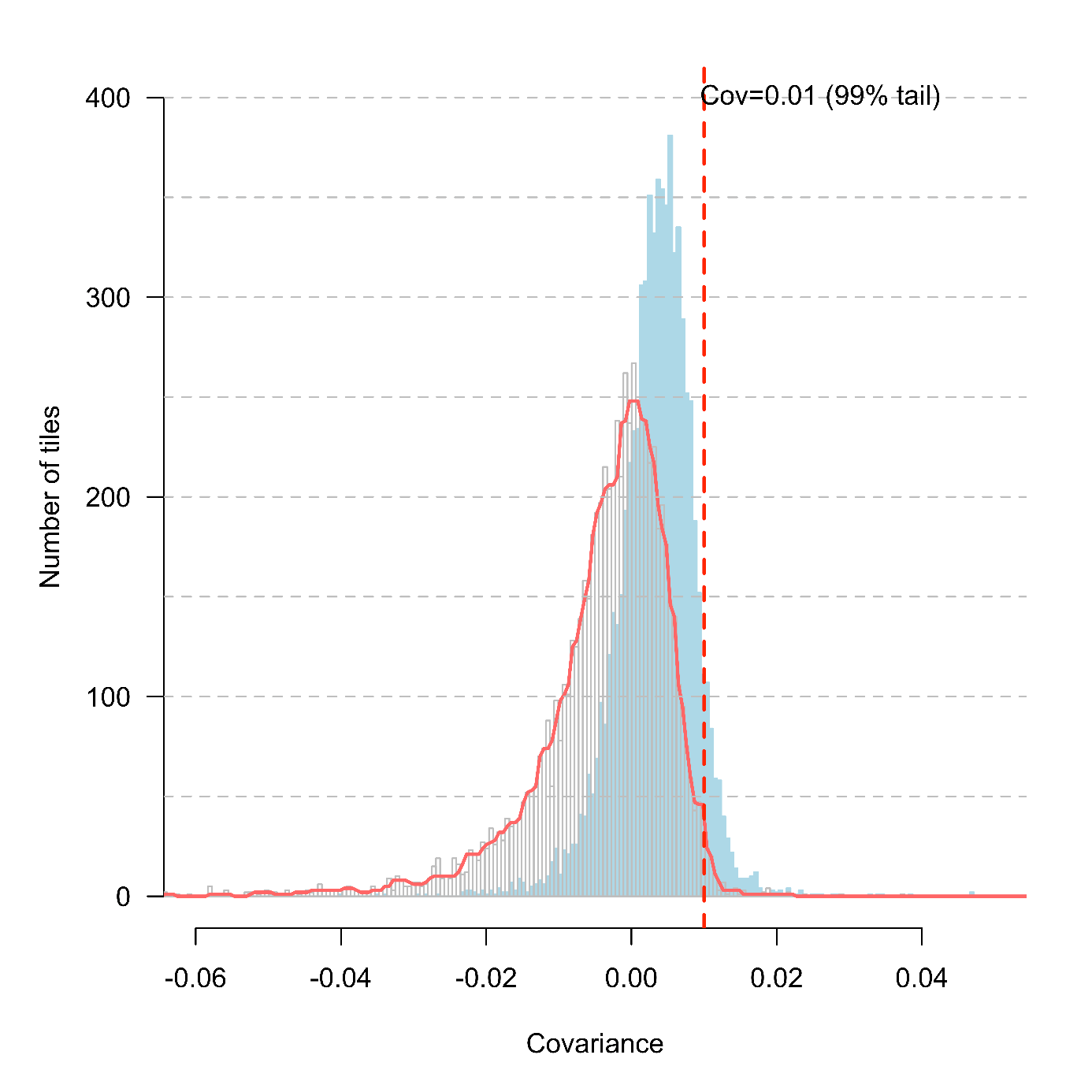
**Figure S4.** Distribution of the temporal covariances between allelic frequency changes between the two oldest time periods (*Cov(Δ_1680-1850_, Δ_1850-1960_))*, calculated for tiles of 100kb.

The distribution corresponds to the covariances calculated at the tile level. The red distribution corresponds to the minimum values between the three forests ; the red dotted cut off line (0.01) is the threshold used to select the outliers tiles (104 tiles which represent 1% of the tiles). The blue shaded distribution corresponds to the mean covariances across the three forests.
