# Supplementary material for: Genome‐wide evolutionary response of European oaks during the Anthropocene
Source: Evol Lett. 2022 Jan 5;6(1):4–20. doi: 10.1002/evl3.269 (PMC8802238; doi:10.1002/evl3.269)

**Figure S5.** Manhattan plot of the temporal covariances of allelic frequency changes between the two earliest time periods (Cov(Δ_1680-1850_, Δ_1850-1960_)) and of Fst between the cohorts delimiting the time periods (Fst_1680-1850_ and Fst_1850-1960_). Covariance and Fst values were calculated at the tile level. Black and grey dots correspond to the covariance and Fst values for each tile within each forest. Red bars are outlier tiles for which the covariances within a forest are larger than 0.01, which was the threshold used for identifying outlier tiles (see text).

**Bercé**


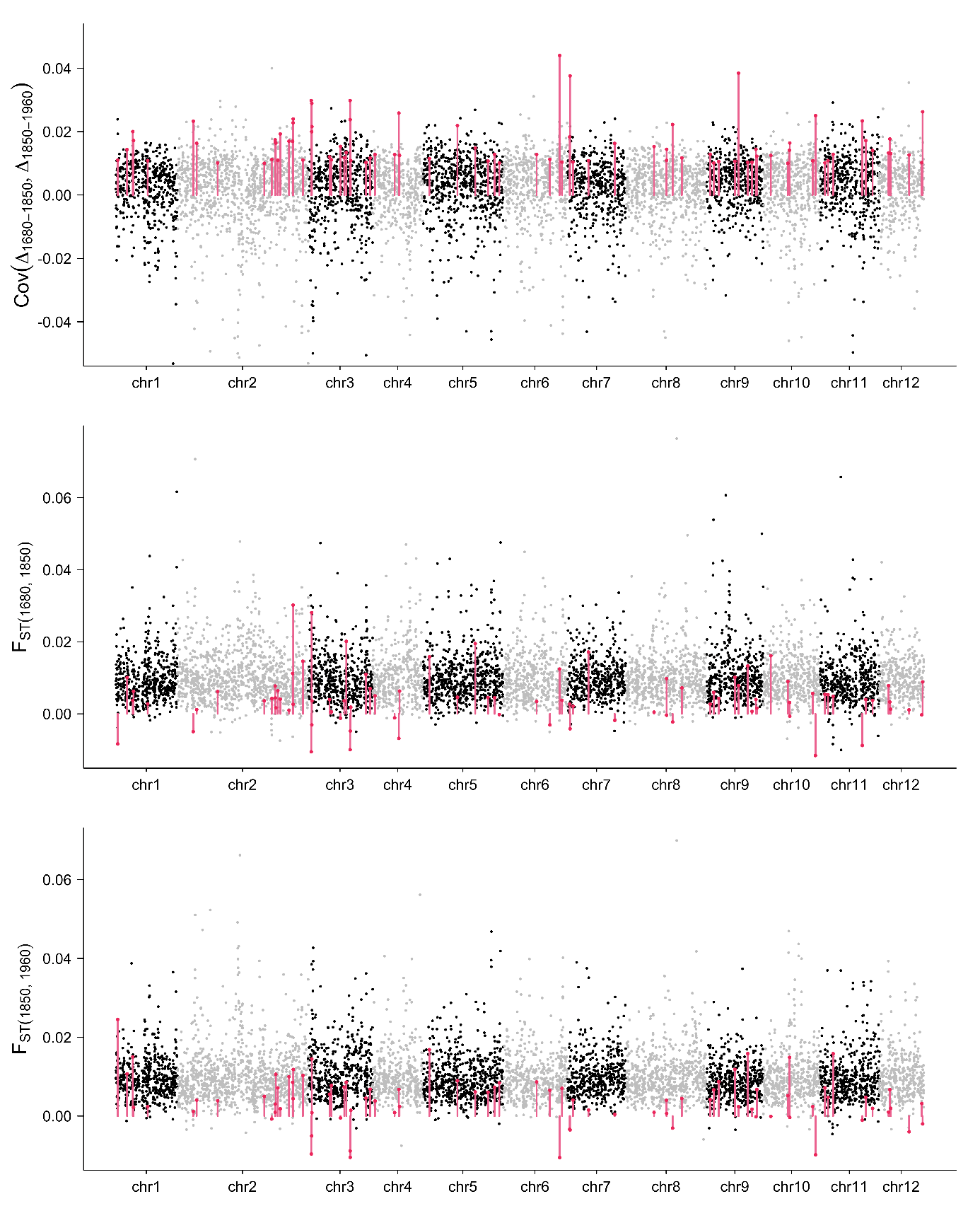


**Réno Valdieu**


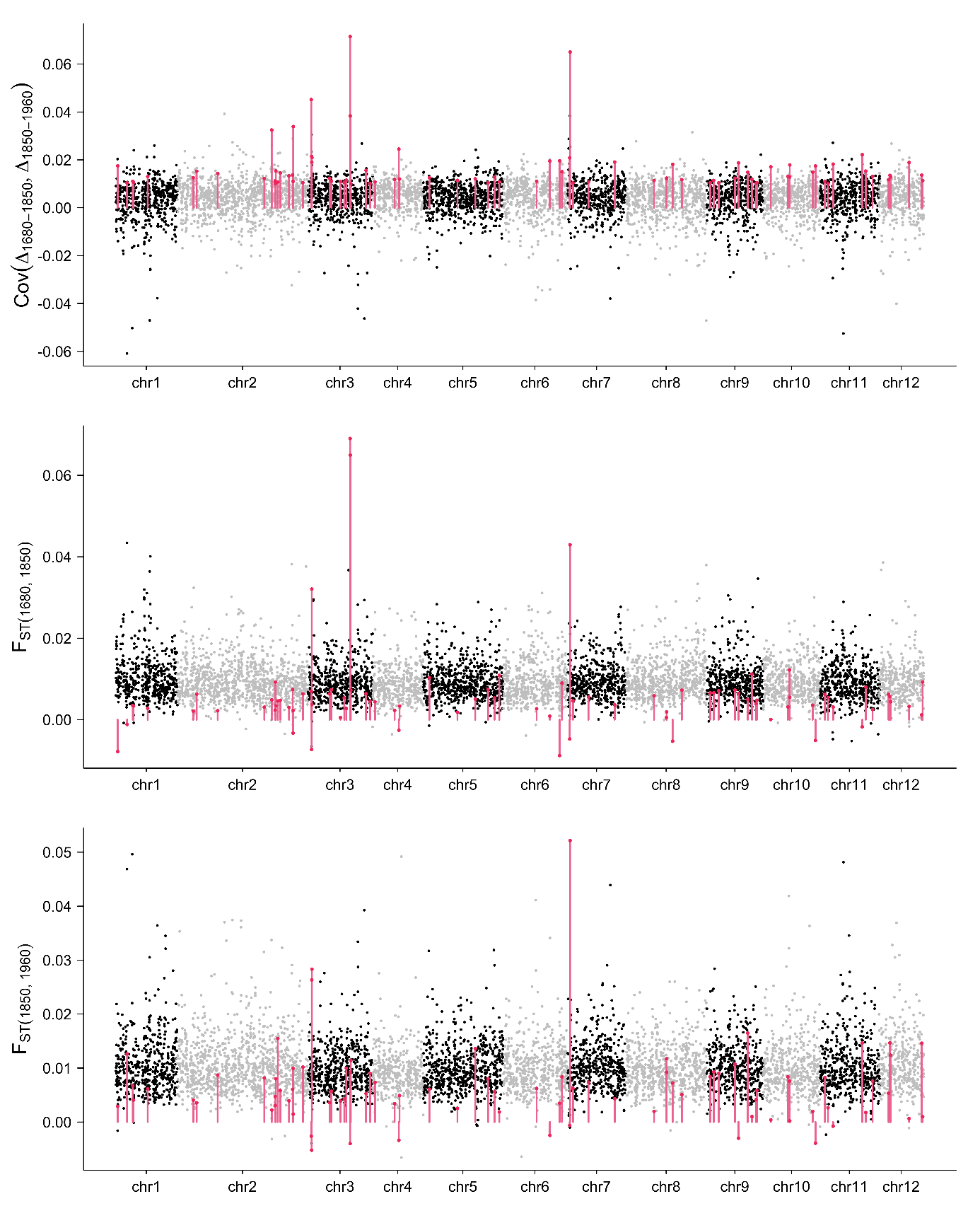


**Tronçais**


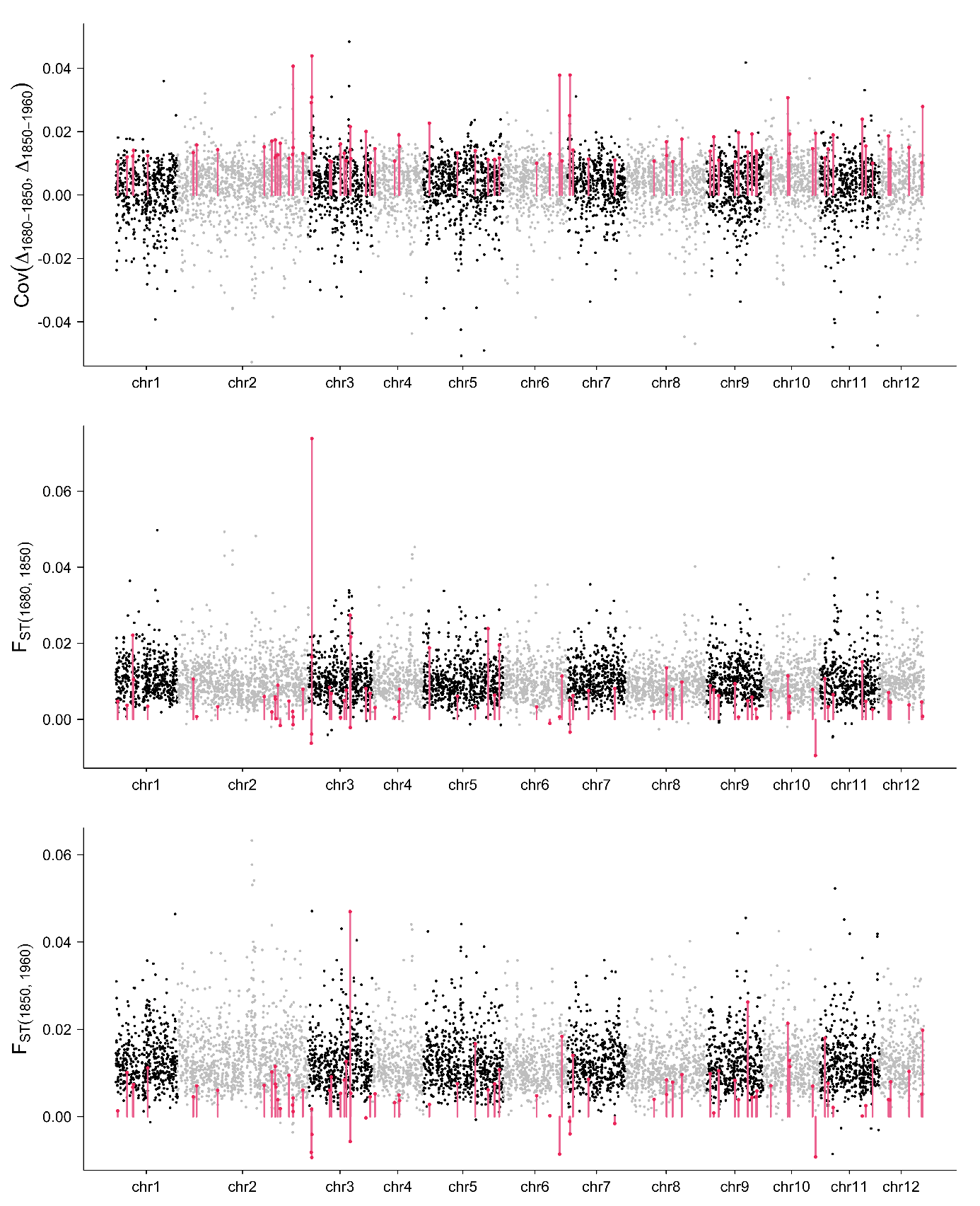

Supplement: Supplementary file 5 — Figure S5. Manhattan plot of the temporal covariances of allelic frequency changes between the two earliest time periods (Cov(Δ1680‐1850, Δ1850‐1960)) and of Fst between the cohorts delimiting the time periods (Fst1680‐1850 and Fst1850‐1960). [file EVL3-6-4-s010.docx]
