# Supplementary material for: Genome‐wide evolutionary response of European oaks during the Anthropocene
Source: Evol Lett. 2022 Jan 5;6(1):4–20. doi: 10.1002/evl3.269 (PMC8802238; doi:10.1002/evl3.269)
Supplement: Supplementary file 6 — Figure S6 Biological network showing the 15 selected Cell Processes and their 74 connected Arabidopsis genes (according to the pathway Studio™Plant database). [file EVL3-6-4-s004.docx]

**Figure S6**

Biological network showing the 15 selected Cell Processes and their 74 connected *Arabidopsis* genes (according to the pathway Studio™Plant database).
Genes putatively involved in Cell Processes related to biotic/abiotic responses are highlited in orange.
