# Supplementary material for: Genome‐wide evolutionary response of European oaks during the Anthropocene
Source: Evol Lett. 2022 Jan 5;6(1):4–20. doi: 10.1002/evl3.269 (PMC8802238; doi:10.1002/evl3.269)
Supplement: Supplementary file 7 — Table S1. Geographic coordinates of sampled forests and age structured cohorts [file EVL3-6-4-s007.docx]

| Forest | Cohort | Compartment# | Latitude | Longitude | Variation elevation (m) |
| --- | --- | --- | --- | --- | --- |
| **Bercé**  (5405ha)  Lat :47.8092  Long :0.4004 | B1 | 227 | 47.78582 | 0.48701 | 161 to 173 |
|  | B2 | 157 | 47.80993 | 0.39948 | 171 to 179 |
|  | B3 | 255 | 47.77766 | 0.49389 | 151 to 161 |
|  | B4 | 226 | 47.78672 | 0.49221 | 155 to 171 |
| **Réno-Valdieu**  (1645ha)  Lat :48.5146  Long: 0.6706 | R1 | 39 | 48.52466 | 0.67761 | 243 to 258 |
|  | R2 | 38 | 48.52760 | 0.67888 | 240 to 245 |
|  | R3 | 59 | 48.51511 | 0.67110 | 246 to 264 |
|  | R4 | 44-45 | 48.52579 | 0.66189 | 243 to 256 |
| **Tronçais**  (10600ha)  Lat :46.6598  Long:2.7059 | T1 | 152 | 46.67122 | 2.77253 | 243 to 274 |
|  | T2 | 62 | 46.65991 | 2.80044 | 265 to 272 |
|  | T3 | 150 | 46.67754 | 2.78378 | 252 to 267 |
|  | T4 | 234 | 46.65815 | 2.70625 | 227 to 267 |

**Table S1.** Geographic coordinates of sampled forests and age structured cohorts
