# Supplementary material for: Genome‐wide evolutionary response of European oaks during the Anthropocene
Source: Evol Lett. 2022 Jan 5;6(1):4–20. doi: 10.1002/evl3.269 (PMC8802238; doi:10.1002/evl3.269)
Supplement: Supplementary file 13 — Supporting Information S1, S2, S3 [file EVL3-6-4-s001.docx]

**Supporting Information**

**Supporting Information S1**

Even-aged oak forests and age structured cohorts

**Supporting Information S2**

Climatic trends over the studied period in the three forests.

**Supporting Information S3**

Enrichment analysis of genes located in outlier tiles.

**Supporting Information S1**

**Even-aged oak forests and age structured cohorts**

Most sessile oak stands in France are managed under even-aged regimes (Jarret 2004). Under such regimes, a forest is subdivided in a number of parcels (compartments) comprising trees that are approximately the same age, and all age classes are evenly distributed in different compartments. We sampled three forests located in the western part of France (Tronçais, Bercé and Réno-Valdieu (Figure 1A), where the rotation age is about 200 years and thus comprising compartments of different ages from 0 to 200 years. However within each of these forests, foresters have also maintained “older” age class compartments for patrimonial and historical reasons. In particular compartments contemporary of the first implementation of even-aged regimes that took place in the mid 17^th^ century by the statesman Colbert were maintained (Gallon 1752). We sampled 4 compartments in each forest that were approximatively 340, 170, 60 and 12 years old (born approximatively in 1680, 1850, 1960 and 2008), which we will later call age structured cohorts (Table S1, Figure 1B). The area of a compartment varies between 10 to 30 ha (Figure1B) and thus comprises census sizes of about 2000 to 6000 trees for the oldest cohorts. These numbers increase for younger cohorts and may reach up 1*10^6^ to 3*10^6^ in the youngest stages. On average, compartments corresponding to the different cohorts were separated by less than 2 kms (Figure 1A) and were also located at similar elevations (Table S1), thus limiting microenvironmental differentiation between cohorts, and maintaining potential gene flow among cohorts. All sampled trees were georeferenced, and an additional limited number of trees were felled for age assessments (Figure 1B). Dendrochronological age assessments matched with management documents. The period of establishment of a cohort was about 30 years for the oldest cohorts, which corresponds to the time lag that was necessary for renewing a stand by natural regeneration. In more recent times regenerations durations extended over 10 to 20 years (Figure 1B).

**Supporting Information S2**

**Climatic trends over the studied period in the three forests.**

Temperature reconstructions of the last millennium indicate substantial changes (Corona *et al.* 2010; Luterbacher *et al.* 2016; Anchukaitis *et al.* 2017; Neukom *et al.* 2019; Wang *et al.* 2019). A consistent trend towards warmer climates during the Medieval period, followed by a much cooler period between approximately 1450 and 1850 has been reported. This 400 year-long period is described as the “Little Ice Age” (LIA) (Matthes 1939; Tkachuck 1983). Mean summer temperatures decreased by 0.5°C to 2°C between the Medieval Warm Period and the Little Ice Age, and then increased again, by more than 1.5°C, to reach current values (Corona *et al.* 2010; Anchukaitis *et al.* 2017). The causes of the LIA remain a matter of debate (Nesje & Dahl 2003; Crowley *et al.* 2008; Palastanga *et al.* 2011; van Oldenborgh *et al.* 2013; Ruddiman *et al.* 2016; Owens *et al.* 2017), but the impact of this period on agriculture and society at large is clear (Pfister 1984; Fagan 2002; Parker 2013).

We used regional and temporal high-resolution (1 km coarse resolution) temperature reconstructions based on a combination of instrumental data, documentary records and ice core and tree ring proxy data by (Luterbacher *et al.* 2004) to monitor retrospectively temperature changes in the three forests since the mid seventeen century (Figure S1). These data illustrate the temperature trends that have been reported earlier since the LIA. The late LIA was dominated by two harsher periods which occurred during the late seventeen century overlapping with the Maunder Minimum (MM) solar activity (1645-1715, (Owens *et al.* 2017)) and during the mid-nineteenth century (1840 to 1860) a period that was preceded by intense volcanic activity was reported (Crowley *et al.* 2008; Bronnimann *et al.* 2019) (Figure S1).

There is also observational evidence of an increased frequency of extreme cold winters during these two periods based on instrumental temperatures recorded at the Observatory of Paris between 1676 and 2010 (Rousseau 2012) (Figure S2, Figure 3D). Occurrences of extreme winters during that period are also supported by historical documents. In his seminal work Le Roy Ladurie mentions that 1690 -1700 was the coldest decade in modern history with extreme winters occurring in 1695 to 1698 ((Le Roy Ladurie 2004), p.437, 487) and 1708-1709 is the worst winter ever reported ( (Le Roy Ladurie 2004) p.514 therein; (Luterbacher *et al.* 2004)). Similarly the decade 1840-1850 is a period where 3 extreme winters (1841, 1845, 1847 in addition to 1830 and 1838) were reported in Rousseau’s survey (Rousseau 2012).

Similarly to extreme winters we examined also occurrences of extreme summer droughts by mining Cook’s data base of Old World megadroughts (Cook *et al.* 2015). This data base provides hydroclimatic reconstructions based on instrumental, historical, archaeological, and natural records during the last millennium. The data base allows to draw year to year regional maps of Palmer Drought severity indices (Palmer 1965; van der Schrier *et al.* 2013) (Figure S3, Figure 3D). Occurrences of extreme summer droughts mirror to some extent also occurrences of extreme winters, thus confirming that the LIA was not just a cooler period but also an age of climatic extremes (Fagan 2002; Le Roy Ladurie 2004, 2006; Blom 2019). While there was a clear overlap of extreme summers and winters during the two most severe periods of the LIA (late seventeenth century and mid-nineteenth century, Figure 3D), the overall frequency of extreme events decreased notably after 1850. The two oldest age structured cohorts are almost synchronous to the two most severe periods recorded during the LIA. Cohort 4 in the three forest originated during the late seventeenth century while trees of cohort 3 established in the mid-nineteenth century (Figure 3D, Figure S2 and S3).

**Supporting Information 3**

**Enrichment and network analysis of genes located in outlier tiles of covariances between the two earliest time periods** $\mathbf{Cov}\boldsymbol{(\Delta}_{\mathbf{1680-1850}}\mathbf{,}\boldsymbol{\Delta}_{\mathbf{1850-1960}}\mathbf{)}$**.**

Enrichment analysis identified several Biological Processes (BP), most of which are related to the “plant-type hypersensitive response”, “defense response to fungus”, “wax and cutin biosynthetic processes” and “anther dehiscence”, with higher connectivity between the two first terms which gather 15 and 13 genes respectively (Figure 7, Table S3). Fifteen genes belonged to the group “defense response to fungus”, eight of which encode proteins similar to AT2G34930, a LRR disease resistance family protein, one was similar to AT3G59660 (BAGP1) required for fungal resistance, one was similar to AT5G64120 (PRX71) encoding a cell wall-binding peroxidase involved in lignification and important for fungal defense, two genes similar to AT3G51550 (FER), a plasma membrane-localized receptor-like kinase involved in fungal infection but also in flowering regulation, and three genes similar to AT1G02205 (CER1), which is associated with the production of stem epicuticular wax and pollen fertility. These multiple copies of *Arabidopsis* homologs correspond to tandem duplications located on chromosomes 3 (AT2G34930, 8 homologs), chromosome 12 (AT3G51550 (FER), 2 homologs) and on the unassigned scaffold Qrob_H2.3_Sc0000124 for AT1G02205 (CER1) (3 homologs), respectively (Table S2). “Cutin biosynthetic process” included four genes: two genes similar to AT4G00400 (GPAT8) and two other similar to AT2G38110 (GPAT6). Cutin is a polymer that is partly covered and interspersed with waxes to form the cuticle, a physical barrier that protects the plant against water loss, irradiation, xenobiotics, and pathogens (Serrano *et al.* 2014).

A total of four genes were associated with the “Anther dehiscence” GO term: one was similar to AT2G02970 (APY6) involved in pollen exine pattern formation and anther dehiscence, and three similar to AT3G13890 (MYB26), which is known to control cellulosic secondary wall thickening in the endothecium. Only two of the three copies were tandem duplicates.

The term “ADP binding” was the most significant Molecular Function ontology group recognized in the enrichment analysis (Table S4). Most of the genes in this category encode proteins carrying NB-ARC or LRR domains and one transmembrane receptor, including the 12 oak homologs of RPM1 and RPP13 described above. This finding is consistent with the importance of the nucleotide binding site (NBS) and ATP/ADP binding in pathogen sensing(DeYoung & Innes 2006). Finally, “extrinsic component of plasma membrane” is the most significant Cellular Component GO terms (Table S5). It gathers the 12 oak homologs of RPM1, consistent with previous results.

In addition to the enrichment analysis, biological relationships between the identified 280 oak protein-coding genes were also retrieved using Pathway Studio™ Plant knowledgebase. From the 250 oak genes mapped to *Arabidopsis* homologs, 143 mapped to 101 Arabidopsis genes but without cellular processes information (data not shown) and 107 mapped to 74 *Arabidopsis* genes were involved in at least one cellular process (Table S6). A total of 923 relations were found between 378 cellular processes and the 74 Arabidopsis genes (Table S6). It is very difficult to visualize a network of 987 entities (74 genes and 378 processes) with 923 relationships. For the sake of clarity, we reduced this network to the 15 most connected cellular processes (Figure S6). The reduced network of Figure S6 includes about 60% of the *Arabidopsis* and oak genes (45 and 66 genes respectively), and provides therefore an aggregated view of the most represented cellular processes. Genes of the reduced network illustrated in Figure S6 and highlighted in orange,  potentially involved in either biotic or abiotic resistance mechanisms, represent about 31% of *the Arabidopsis* genes (23/74) and 40% of the oak genes (43/107).

**References**

Anchukaitis, K.J., Wilson, R., Briffa, K.R., Buntgen, U., Cook, E.R., D'Arrigo, R. *et al.* (2017). Last millennium Northern Hemisphere summer temperatures from tree rings: Part II, spatially resolved reconstructions. *Quaternary Science Reviews*, 163, 1-22.

Blom, P. (2019). *Nature's mutiny: How the LIttle Ice Age of the long seventeenth century transformed the west and shaped the present.* LIveright, New York.

Bronnimann, S., Franke, J., Nussbaumer, S.U., Zumbuhl, H.J., Steiner, D., Trachsel, M. *et al.* (2019). Last phase of the Little Ice Age forced by volcanic eruptions. *Nature Geoscience*, 12, 650-+.

Cook, E.R., Seager, R., Kushnir, Y., Briffa, K.R., Buntgen, U., Frank, D. *et al.* (2015). Old World megadroughts and pluvials during the Common Era. *Science Advances*, 1.

Corona, C., Guiot, J., Edouard, J.L., Chalie, F., Buntgen, U., Nola, P. *et al.* (2010). Millennium-long summer temperature variations in the European Alps as reconstructed from tree rings. *Climate of the Past*, 6, 379-400.

Crowley, T.J., Zielinski, G., Vinther, B., Udisti, R., Kreutz, K., Cole-Dai, J. *et al.* (2008). Volcanism and the Little Ice Age. *Pages News*, 16, 22-23.

DeYoung, B.J. & Innes, R.W. (2006). Plant NBS-LRR proteins in pathogen sensing and host defense. *Nature Immunology*, 7, 1243-1249.

Fagan, B. (2002). *The little ice age. How climate made history (1300-1850)*. Basic Books, New York

Gallon, D. (1752). *Conférence de l'ordonnance de Louis XIV du mois d'Aout 1969 sur le fait des Eaux et Forêts*. Brunet, Paris.

Jarret, P. (2004). *Chênaie atlantique*. Lavoisier.

Le Roy Ladurie, E. (2004). *Histoire humaine et comparée du climat. Canicules et glaciers XIIIième-XVIIIième siècles.* Fayard, Paris.

Le Roy Ladurie, E. (2006). *Histoire humaine et comparée du climat. Disettes et révolutions 1740-1860.* Fayard, Paris.

Luterbacher, J., Dietrich, D., Xoplaki, E., Grosjean, M. & Wanner, H. (2004). European seasonal and annual temperature variability, trends, and extremes since 1500. *Science*, 303, 1499-1503.

Luterbacher, J., Werner, J.P., Smerdon, J.E., Fernandez-Donado, L., Gonzalez-Rouco, F.J., Barriopedro, D. *et al.* (2016). European summer temperatures since Roman times. *Environmental Research Letters*, 11.

Matthes, R.E. (1939). Report of the committe on glaciers. *Transactions of the American Geophysiscal Union*, 20, 518-523.

Nesje, A. & Dahl, S.O. (2003). The 'Little Ice Age'-only temperature ? *The Holocene*, 13, 139-145.

Neukom, R., Barboza, L.A., Erb, M.P., Shi, F., Emile-Geay, J., Evans, M.N. *et al.* (2019). Consistent multidecadal variability in global temperature reconstructions and simulations over the Common Era. *Nature Geoscience*, 12, 643-+.

Owens, M.J., Lockwood, M., Hawkins, E., Usoskin, I., Jones, G.S., Barnard, L. *et al.* (2017). The Maunder minimum and the Little Ice Age: an update from recent reconstructions and climate simulations. *Journal of Space Weather and Space Climate*, 7.

Palastanga, V., van der Schrier, G., Weber, S.L., Kleinen, T., Briffa, K.R. & Osborn, T.J. (2011). Atmosphere and ocean dynamics: contributors to the European Little Ice Age? *Climate Dynamics*, 36, 973-987.

Palmer, W.C. (1965). *Meteorological drought*. US Department of Commerce, Weather Bureau, Washington.

Parker, G. (2013). *Global crisis. War, climate change and catastrophe in the seventeenth century.* Yale University Press, New Haven.

Pfister, C. (1984). *Das Klima der Schweiz von 1525-1860 und seine Bedeutung in der Geschichte von Bevölkerung und Landwitrschaft. Volume 2. Bevölkerung, Klima und Agrarmodernisierung 1525-1860*. Paul Haupt, Bern.

Rousseau, D. (2012). Identification des grands hivers de 1676 à 2010 à l'aide de séries thermométriques de Paris. In: *Canicules et froids extrêmes* (eds. Berchtold, J, Le Roy Ladurie, E, Sermain, J-P & Vasak, A). Hermann Paris, pp. 345-360.

Ruddiman, W.F., Fuller, D.Q., Kutzbach, J.E., Tzedakis, P.C., Kaplan, J.O., Ellis, E.C. *et al.* (2016). Late Holocene climate: Natural or anthropogenic? *Reviews of Geophysics*, 54, 93-118.

Serrano, M., Coluccia, F., Torres, M., L'Haridon, F. & Metraux, J.P. (2014). The cuticle and plant defense to pethogens. *Frontiers in Plant Science*, 5, 274.

Tkachuck, R.D. (1983). The Little Ice Age. *Origins*, 10, 51-65.

van der Schrier, G., Barichivich, J., Briffa, K.R. & Jones, P.D. (2013). A scPDSI-based global data set of dry and wet spells for 1901-2009. *Journal of Geophysical Research-Atmospheres*, 118, 4025-4048.

van Oldenborgh, G.J., de Laat, A.T.J., Luterbacher, J., Ingram, W.J. & Osborn, T.J. (2013). Claim of solar influence is on thin ice: are 11-year cycle solar minima associated with severe winters in Europe? *Environmental Research Letters*, 8.

Wang, Z.Y., Wang, J.L. & Zhang, S.J. (2019). Variations of the global annual mean surface temperature during the past 2000 years: results from the CESM1. *Theoretical and Applied Climatology*, 137, 2877-2887.
